# Cell-permeable capsids as universal antigen carrier for the induction of an antigen-specific CD8^+^ T-cell response

**DOI:** 10.1038/s41598-017-08787-0

**Published:** 2017-08-29

**Authors:** Sami Akhras, Masako Toda, Klaus Boller, Kiyoshi Himmelsbach, Fabian Elgner, Marlene Biehl, Stephan Scheurer, Meike Gratz, Stefan Vieths, Eberhard Hildt

**Affiliations:** 1 Department of Virology, Paul-Ehrlich-Institut, 63225 Langen, Germany; 2 Department of Allergology, Paul-Ehrlich-Institut, 63225 Langen, Germany; 3 Department of Immunology, Paul-Ehrlich-Institut, 63225 Langen, Germany; 4 German Center for Infection Research (DZIF), 38124 Braunschweig, Germany

## Abstract

Vaccine platforms that can be flexibly loaded with antigens can contribute to decrease response time to emerging infections. For many pathogens and chronic infections, induction of a robust cytotoxic T lymphocytes-mediated response is desirable to control infection. Antigen delivery into the cytoplasm of antigen presenting cells favors induction of cytotoxic T cells. By fusion of the cell-permeable translocation motif (TLM)-peptide to the capsid-forming core protein of hepatitis B virus, and by insertion of the strep-tag in the spike tip (a domain that protrudes from the surface of the capsid), cell-permeable carrier capsids were generated that can be flexibly loaded with various antigens. Loading with antigens was demonstrated by electron microscopy, density gradient centrifugation and surface plasmon resonance spectroscopy. Confocal immunofluorescence microscopy showed that cell-permeable carrier capsids mediate transfer of cargo antigen into the cytoplasm. Using cell-permeable carrier capsids loaded with ovalbumin as model antigen, activation of antigen presenting cells and ovalbumin-specific CD8^+^ T-cells, which correlates with enhanced specific killing activity, was found. This demonstrates the capacity of TLM-carrier-capsids to serve as universal antigen carrier to deliver antigens into the cytoplasm of antigen presenting cells, which leads to enhanced MHC class I-mediated presentation and induction of antigen-specific cytotoxic T lymphocytes response.

## Introduction

Vaccination is one of the most effective means to combat infectious diseases. Infections by new emerging pathogens such as Ebola or Zika virus that can rapidly reach epidemic levels require a concept for the rapid development of vaccines. In addition there are a variety of infectious diseases or chronic infections that cannot be efficiently controlled by an exclusively B-cell driven approach. Therefore, it would be desirable to have a strategy for inducing a cytotoxic T lymphocytes (CTL)-mediated immune response as an additional branch of the immune response.

Vaccine platforms are an important tool to shorten response time on emerging pathogens. Well established and characterized vaccine platforms are for example modified vaccinia virus Ankara (MVA), vesicular stomatitis virus (VSV) or adenovirus that enable the limited expression of the antigen of interest in the context of the viral genome. In principle, the use of replication incompetent or attenuated viral genomes ensures the controlled expression of the antigen for a limited period^1–3^.

In addition, virus-like particles (VLPs) can be used as a vaccine platform for direct delivery of the antigen. The highly ordered VLP structure enables the presentation of the foreign antigens in a repeated and condensed pattern which facilitates the induction of a robust humoral B-cell response^4–6^.

The hepatitis B virus (HBV) capsid is a structurally well characterized VLP and is being widely investigated as a vaccine template^7^. The hepatitis B virus capsid (core) is assembled by either 120 dimers (*T* = 4 symmetry) or 90 dimers (*T* = 3 symmetry) of the hepatitis B core antigen (HBcAg). The prominent structure formed by the HBcAg dimers is the spike tip that protrudes out of the surface of the assembled capsid^8^. In between amino acid (aa) 78 and 83 of the HBV core protein, the spike tip can be opened and foreign sequences such as GFP can be inserted without affecting the assembly to particles^9–11^. However, the size of the inserted protein is limited and some structural requirements must be fulfilled: i.e. the distance between the N- and C-terminal domains of the inserted protein must fit in the opened spike tip^12^. To overcome this obstacle, alternatives to the direct insertion can be applied such as the chemical coupling of the foreign antigens to the capsid surface^13^. In our study, we adress this problem by inserting strep-tag in the spike tip region which allows the coupling of foreign antigens, produced as streptavidin fusion peroteins, on the surface of the capsids, and that allows the use of the capsid as a universal antigen carrier. A similar strategy was applied previousely by coupling a streptavidin fusion proteins to the surface of biotinylated VLPs^14^. In addition, other coupling systems, such as the covalet bond-forming tag/domain, the SpyTag/SpyCatcher^15, 16^, can be applied as well.

The highly ordered structure of the HBV capsid favors the induction of a robust B-cell response by HBV capsid-based VLPs^17, 18^. However, in case of many pathogens or chronic infections the induction of a robust cytotoxic T-cell response in addition to the robust B-cell response is desired to prevent the establishment of an infection or to eliminate a chronic infection. Induction of a specific cytotoxic T lymphocytes (CTLs) response requires the activation of dendritic cells (DCs) and MHC class I-dependent presentation of antigen-specific peptides on the DCs. This can be achieved by different ways such as antigen expression in DCs, or the use of VLPs to deliver the foreign antigens into the DCs^19^. VLPs, including HBcAg-derived VLPs, were proven to be powerful tools to induce a potent CTLs response for vaccination against pathogens and tumors^6, 20–29^. This might be explained by the ability of VLPs to specifically target the DCs^30^. After internalization by the professional antigen presenting cells (APCs), particularly DCs, the endocytosed VLPs are processed and presentation of the epitopes occurs after cross-presentation by the MHC class I molecules for the induction of CTLs response^31–33^. Moreover, VLPs (including HBV capsid-derived VLPs) were shown to induce upregulation of co-stimulatory molecules and/or cytokines secretion in DCs^34, 35^ which might additionaly account for the high immunogenicity of VLPs. As an alternative of the endocatotic pathway, antigens can be directly delivered into the cytoplasm of DCs, followed by (immune-) proteasomal processing, finally leading to MHC class I-dependent presentation of antigen-specific peptides on the surface of DCs^19^. However, efficient delivery of antigens to the cytoplasm of DCs in a VLP-based vaccine requires the capacity to translocate through the plasma membrane of the DCs. This was achieved by fusing the cell permeable TLM-peptide (translocation motive) to the N-terminus of HBcAg resulting in properly assembled capsids, TLM-capsid, that are decorated with TLM-peptides on their surface^36^.

The cell permeable TLM-peptide encompasses 12 amino acids that form an amphipathic α-helix^37^. The TLM-peptide was identified in the PreS2-domain of the HBV surface protein. Functional TLM-peptides are conserved in the PreS-domain of all hepadnaviruses^37^. Fusion of the TLM to other peptides or proteins enables their translocation across the plasma membrane of living cells in an endocytosis- and energy-independent manner without affecting membrane integrity^37–39^.

Previous work using ovalbumin as model antigen has shown that fusion of the TLM-peptide to ovalbumin renders the TLM-Ova fusion protein cell permeable. TLM-Ova triggers the induction of proliferation and cytotoxicity of Ova-specific CD8^+^ T-cells *in vitro* and *in vivo*. This demonstrates that cell-permeable protein vaccines can improve the cellular immune response^40^.

Due to the presence of the TLM-peptides on their surface, the TLM-capsids are membrane-permeable^36^. If nucleic acids were packaged in the interior of these cell permeable capsids, efficient gene transfer could be achieved even in primary cells such as primary human hepatocytes^36^. This reflects the capacity of TLM-capsids to translocate across plasma membranes and to transport cargo into cells.

This study describes the development of cell-permeable carrier capsids as vaccine platform that can be flexibly loaded with various antigens via an adapter, their structural characterization, and their functional analysis using ovalbumin as model antigen with respect to their capacity to trigger a robust CTL- response. To our knowledge, there is so far no report in the literature that membrane-permeable VLPs were used as platform for antigen delivery into APCs. Based on this, we think that our approach could provide a new concept in this field.

## Results

### Generation of cell-permeable carrier capsids which harbor strep-tag III in the spike tip

**Figure 1:**
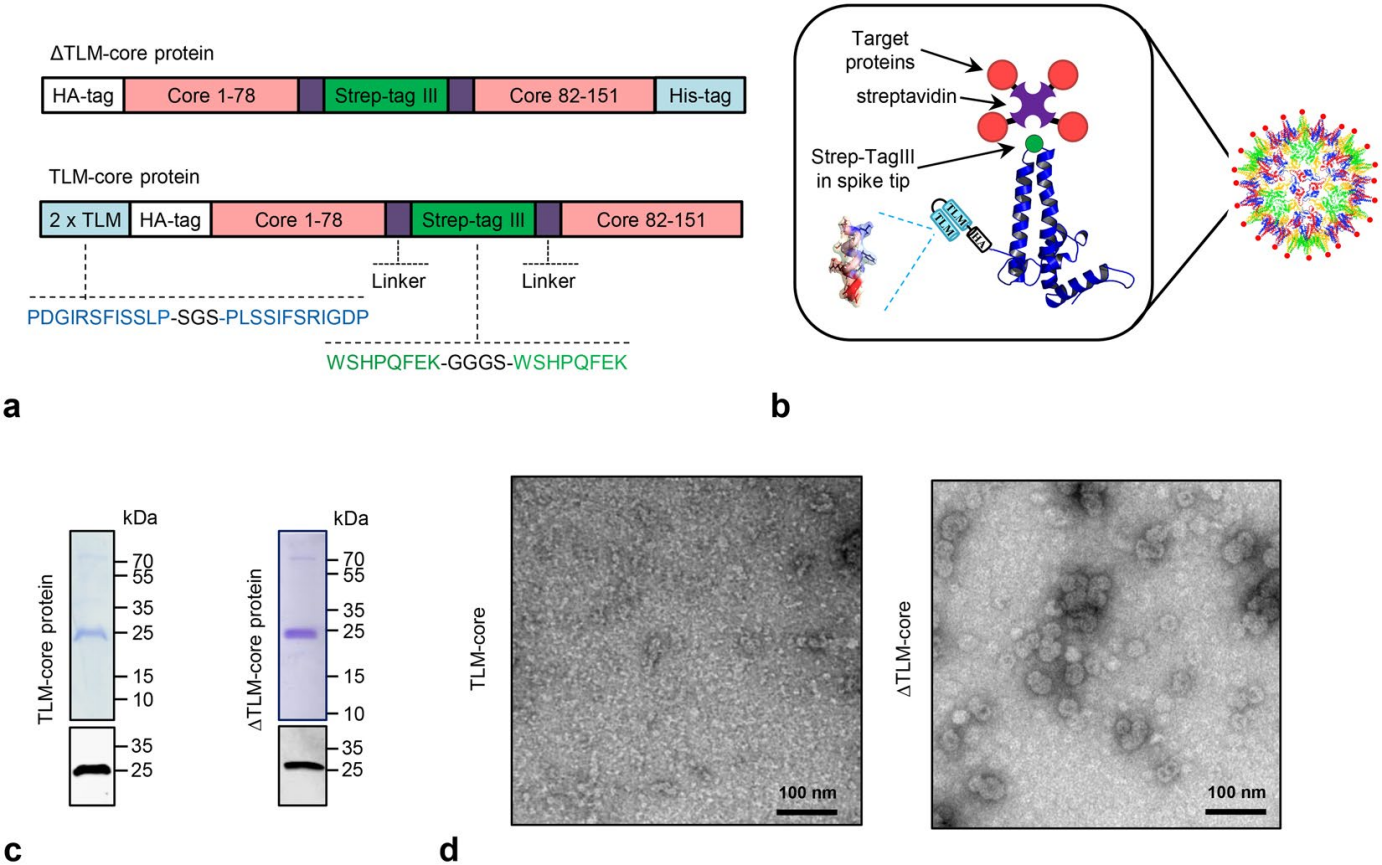
Generation and purification of cell-permeable virus-like particles with strep-tag III in the spike tip (TLM-carrier capsids and ∆TLM-carrier capsid). (**a**) Linear, schematic representation of recombinant HBV-core protein with HA-tag, strep-tag III, and with or without 2xTLM. HA-tag = 9 aa derived from the human influenza hemagglutinin. (**b**) Schematic representation of the TLM-carrier capsid with the target proteins loaded on the surface via streptavidin/strep-tag binding (The schematic representation of the HBV core monomer and particle was modified from Wynne *et al*., 1999^8^). Violet and red parts represent streptavidin and target protein, respectively. (**c**) TLM-core and ∆TLM-core proteins were purified by strep-Tactin affinity chromatography. Selected fractions after the strep-Tactin purification were analyzed by Coomassie stained SDS-gel and Western blotting (see Supplementary Fig. S1). Here elution fractions C10 (for TLM-core) and A3 + A4 (for ∆TLM-core) are shown (coomassie staining: upper panel, Western blot: lower panel). The theoretical molecular weights of TLM-core protein and ΔTLM-core protein are about 25.4 and 24.5 kDa, respectively. (**d**) Transmission electron microscopy (TEM) of the purified TLM-core and ∆TLM-core proteins (negative staining, scale bar = 100 nm).

The generation of cell permeable capsids by fusion of the TLM peptide to the N-terminus of the HBV capsid-forming core protein (HBcAg), which can be used for efficient gene transfer after packaging of DNA in their interior, was previously described^36^. To use these particles as platform for antigen transfer into antigen presenting cells (APCs), a short adapter, based on the strep-tag III, was inserted in the spike tip domain of the TLM-fused HBcAg protein to generate an universal carrier platform that can be flexibly loaded on its surface with variable antigens that are fused to streptavidin (Fig. 1a,b).

**Figure 2:**
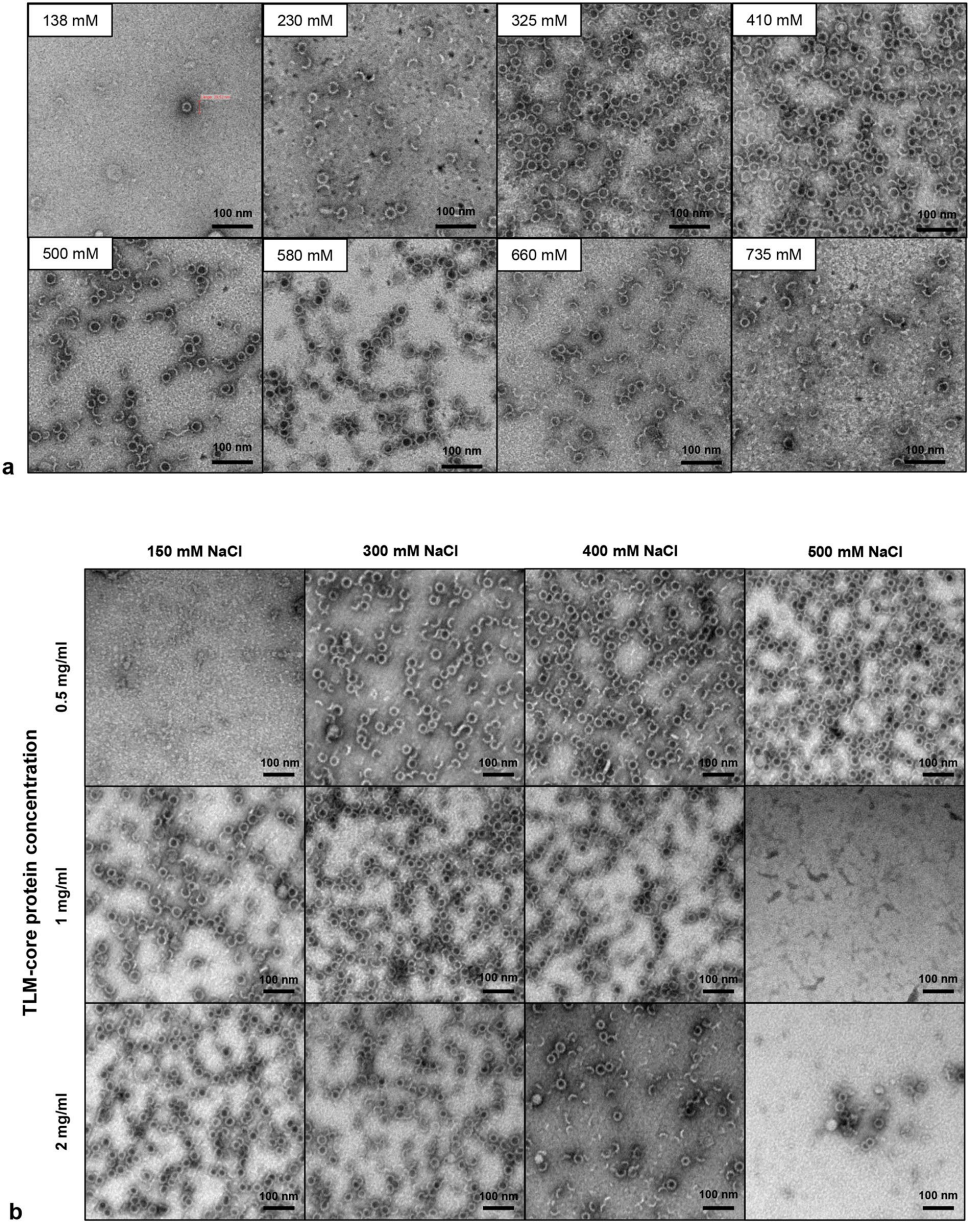
*In vitro* assembly of purified TLM-core protein into TLM-carrier capsids. (**a**) TLM-core protein was produced in *E*. *coli* and purified by strep-Tactin affinity and HiTrap desalting chromatography. Purified protein (150 µg/ml = 5.9 µM) was subjected to *in vitro* assembly by incubating for 24 hours at 0 °C in PBS that contains different NaCl concentrations (138 mM – 735 mM). The assembly products were scanned by TEM. (**b**) Assembly of the purified TLM-core protein was performed at three different protein concentrations (0.5 mg/ml = 19.7 µM, 1 mg/ml = 39.4 µM, and 2 mg/ml 78.7 µM) for 24 hours at 0 °C in PBS that contains different NaCl concentrations (150, 300, 400, and 500 mM). The assembly products were scanned by TEM (negative staining, scale bar = 100 nm). See also Supplementary Fig. S2.

**Figure 3:**
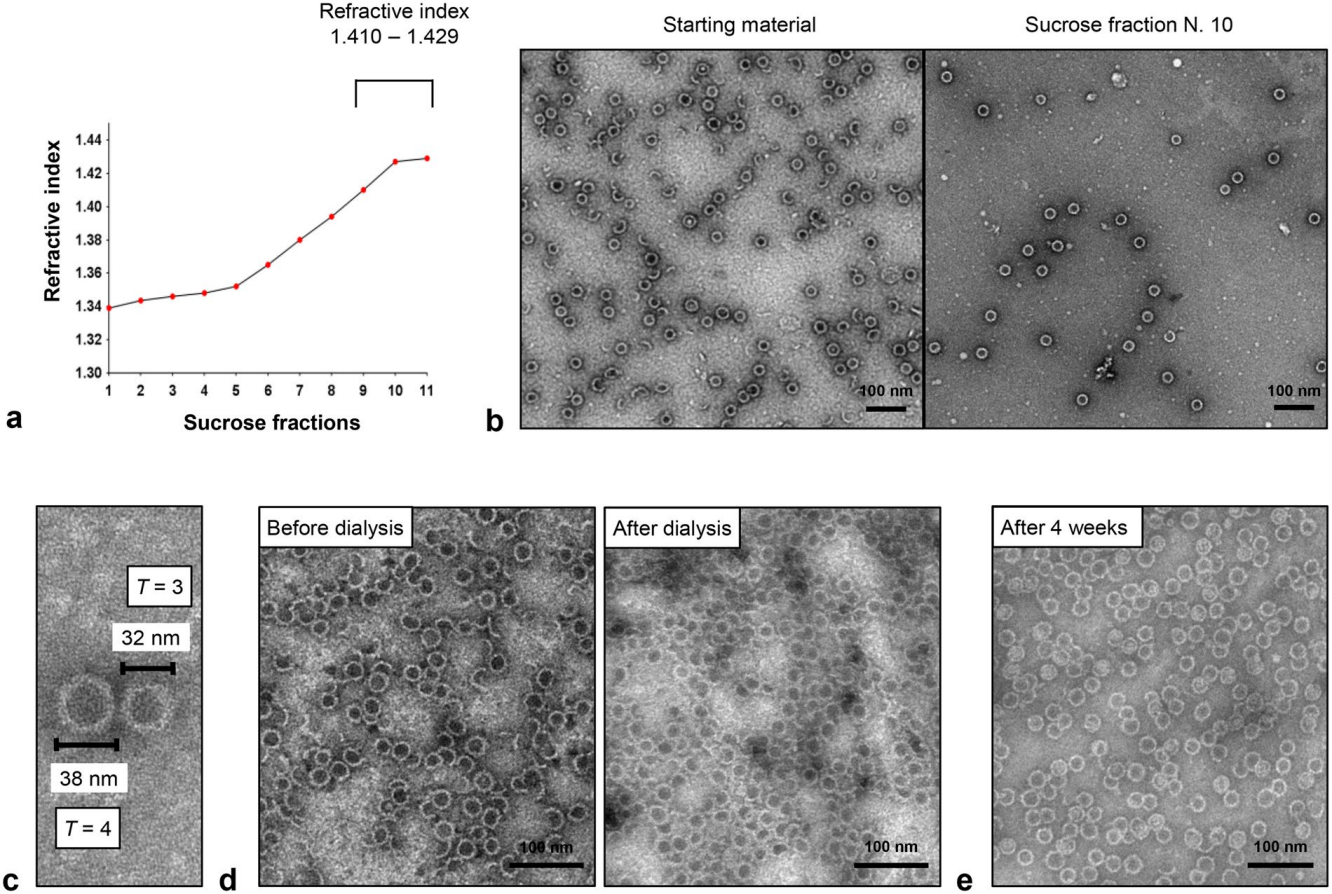
Stability of the assembled TLM-carrier capsids. (**a**) Purified and assembled TLM-carrier capsids (550 µg/ml = 21.6 µM) were laid on discontinuous sucrose gradient (10−70%) and centrifuged for 18 hours at +10 °C, 41000 rpm. Eleven sucrose fractions were collected from the top to the bottom of the tube, and refractive indices were measured. (**b**) TEM pictures (negative staining, scale bar = 100 nm) of the sample before applying on the sucrose gradient (left picture) and after sucrose gradient (fraction number 10, right picture). (**c**) High magnification TEM picture of two particles representing the *T* = 3 and *T* = 4 symmetry. (**d**) The assembly of TLM-core protein (150 µg/ml = 5.9 µM) was performed by increasing NaCl concentration to 325 mM followed by incubating the sample for 24 hours at 0 °C. The sample was then dialyzed against PBS containing 138 mM NaCl for 24 hours at + 4 °C and analyzed by TEM. (**e**) Purified and assembled TLM-carrier capsids (100 µg/ml = 3.9 µM) were incubated for 28 days at +4 °C, and the stability of the assembled core particles was analyzed by TEM. See also Supplementary Fig. S2.

An eubacterial expression vector (pTLMcoreStrep) was generated encoding for a fusion protein that encompasses a tandem TLM followed by an HA-tag and aa 1–78 of the core protein, the strep-tag III replacing aa 79–82 in the spike tip domain and the C-terminal part of the core protein (aa 83–151), subsequently designated as TLM-core. As a control, another eubacterial expression vector (pΔTLMcoreStrep) was generated to produce a similar fusion protein harboring a strep-tag III in the spike tip, but lacks the TLM motives (designated as ΔTLM-core) (Fig. 1a). The recombinant TLM-core and ΔTLM-core proteins were purified from *E*. *coli* expression system by affinity chromatography on a strep-Tactin column (Supplementary Fig. S1). The purity and identity of the purified proteins were demonstrated by SDS-PAGE and Western blot analysis (Fig. 1c and Supplementary Fig. S1). Transmission electron microscopy of the purified proteins revealed that ΔTLM-core protein build properly assembled core particles (Fig. 1d right panel). However, most of the purified TLM-core protein does not represent assembled particles (Fig. 1d left panel). Previous pioneering work from the Zlotnick lab^41^ has carefully characterized the conditions for the *in vitro* disassembly and reassembly of spherical viral capsids, including HBV capsids. Based on this, we tried to trigger the assembly of the purified TLM-core protein into fully assembled TLM-core particles by changing a variety of parameters: Protein concentration, temperature, and NaCl concentration (Fig. 2a,b and Supplementary Fig. S2). An optimal assembly as demonstrated by electron microscopy was achieved at 4 °C for a core protein concentration of 150 µg/ml and an increase of the NaCl concentration up to 325–410 mM (Fig. 2a). The fully assembled TLM-carrier capsids can be further purified and separated from remnants of the partially assembled particles by sucrose density gradient centrifugation as evidenced by electron microscopy (Fig. 3a,b).

Taken together, these data indicate that, based on a eubacterial expression system and subsequent *in vitro* assembly, fully assembled, intact TLM-carrier and ΔTLM-carrier capsids harboring a strep-tag as adapter in the spike tip, can be obtained.

### Assembled TLM-carrier capsids are stable under physiologic conditions

Based on the purified and *in vitro* assembled TLM-carrier capsids, a detailed analysis of their structure and stability was performed. High magnification transmission electron microscopy pictures revealed two different sizes of the particles, about 32 and 38 nm in diameter (Fig. 3c), representing the previously reported *T* = 3 and *T* = 4 variants^8^. Stability under physiologic salt concentrations is an essential issue, if an application as antigen carrier is intended. To investigate this, particles that were *in vitro* assembled in the presence of 325 mM NaCl, were transferred to PBS buffer by dialysis. Subsequent electron microscopy analysis revealed that the stability and integrity of the assembled particles are not affected by shifting the salt concentration to physiologic conditions (Fig. 3d).

To investigate whether freeze/thawing or higher temperatures affect the stability, the assembled TLM-carrier capsid were frozen at −20 °C, or −80 °C and thawed, or incubated at 37 °C for 1 h and analyzed by transmission electron microscopy (TEM). The TEM pictures show that neither freeze/thawing nor incubation at 37 °C affected the stability of the TLM carrier capsids (Supplementary Fig. S2). Moreover, to determine the stability of the particles under long-term storage conditions, the assembled core particles were kept for four weeks at + 4 °C. No changes in the shape and the integrity were observed (Fig. 3e). These results are not surprising since resistance of the HBV capsid to disassociation, after shifting to assembly-unfavorable conditions, and remarkable stability of the assembled HBV capsids were reported previously^42, 43^.

These data demonstrate that the purified TLM-carrier capsids are stable under physiologic salt conditions and can be stored over a longer time period without affecting the integrity of the particles.

### The TLM-carrier capsids can be loaded on their surface with cargo proteins via the strep-tag adapter

**Figure 4:**
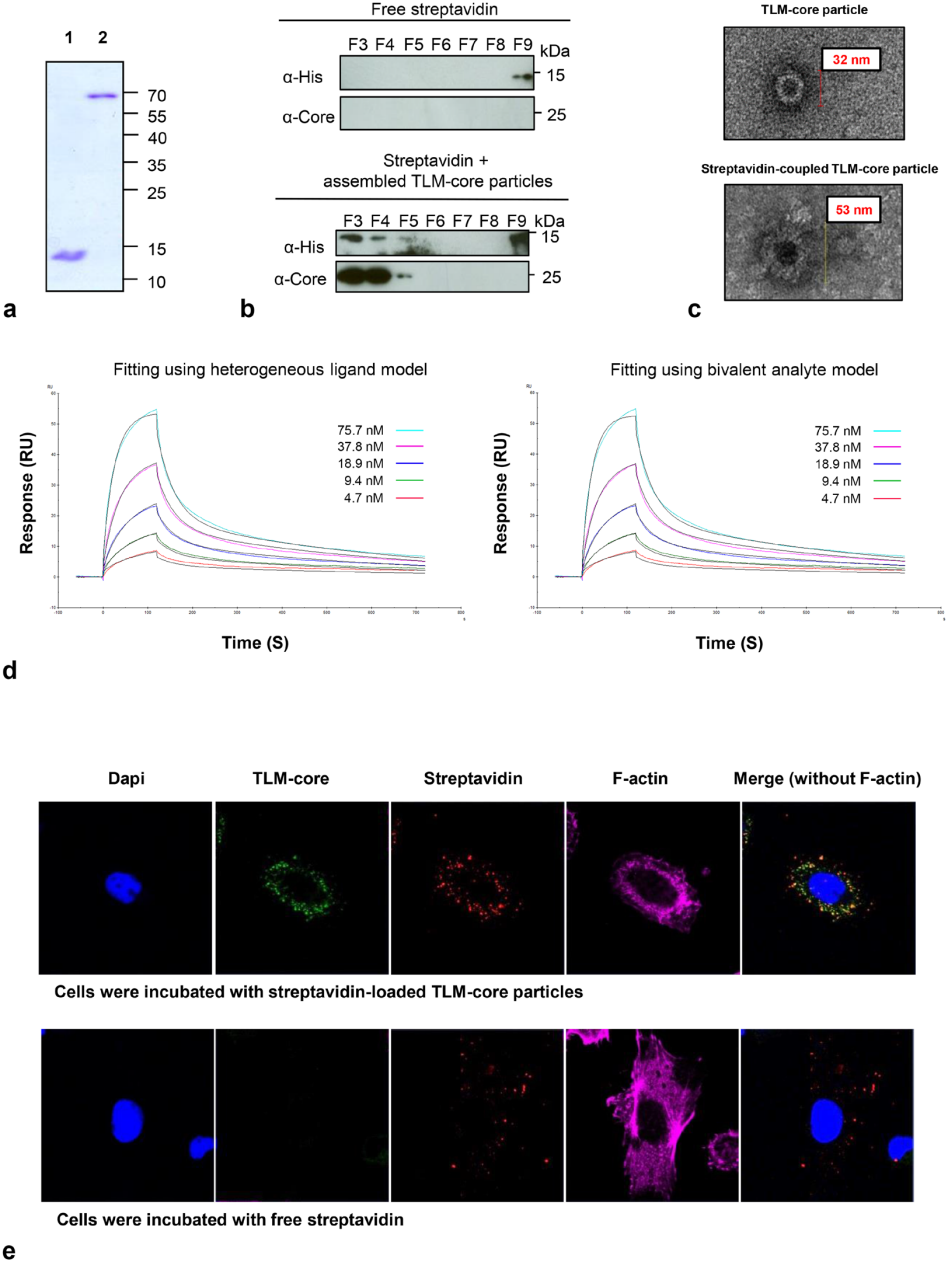
Streptavidin as an adaptor for the coupling on the surface of TLM-carrier capsid. Streptavidin protein was produced in *E*. *coli* and purified under denaturing conditions using Ni-NTA affinity chromatography (see Supplementary Fig. S3). (**a**) Denaturing (lane 1) and non-denaturing (lane 2) SDS-PAGE analysis of the purified and refolded streptavidin (for more details, see *Supplementary Information*). The theoretical molecular weights of streptavidin monomer and tetramer are 14.4 kDa and 57.6 kDa, respectively. (**b**) Binding analysis of streptavidin to the TLM- carrier capsid by sucrose gradient centrifugation (for more details, see material and methods). See also Supplementary Fig. S3. (**c**) High magnification TEM pictures of unloaded TLM-carrier capsid (upper picture) or streptavidin-loaded capsid (lower picture). See also Supplementary Fig. S3. (**d**) Sensorgrams of the surface plasmon resonance (SPR) analysis of the interaction between streptavidin and strep-tag III on the surface of the TLM-carrier capsid. TLM-carrier was immobilized on a CM5-sensor chip and streptavidin was injected in five different concentrations. “Heterogeneous ligand” or “bivalent analyte” fitting models were used. Experimental results are drawn in different colors and theoretical curves in black. For further details, see also Supplementary Fig. S3. (**e**) Confocal laser scanning microscope (CLSM) pictures of Huh 7.5 cells which were incubated with free streptavidin (lower panel) or with TLM-carrier capsid loaded with streptavidin (upper panel). Internalized proteins were stained with HBcAg-specific antibody (green) and streptavidin-specific antibody (red). Actin filaments were stained with TRITC-phalloidin (magenta) and nuclei with DAPI (blue).

The experiments described above demonstrate that highly purified, stable TLM-carrier capsids can be produced. In order to analyze whether the inserted strep-tag III indeed serves as functional adapter that enables loading of these TLM-carrier capsids with antigen, the loading with streptavidin was investigated. Recombinant hexa-his-streptavidin was isolated from a eubacterial expression system by affinity chromatography on a Ni-chelating column under denaturing conditions and refolded (Supplementary Fig. S3). The functionality of the highly purified refolded streptavidin was demonstrated by its capacity to form tetramers under non-denaturing conditions (Fig. 4a) and to bind FITC-biotin (Supplementary Fig. S3). In addition, the purified streptavidin (150 µg) was added to highly purified and assembled TLM-carrier capsid (250 µg). The mixture was loaded on a sucrose gradient (10−80%). As a control, free streptavidin was loaded on the gradient. If the inserted strep-tag III is functional, streptavidin should bind to TLM-carrier capsids and co-migrate with the carrier into the gradient. Western blot analysis of the fractions collected from the gradient using streptavidin- and core-specific antibodies demonstrated that streptavidin co-migrates with the core protein to the high density sucrose fractions, while free streptavidin floated on the top of the gradient (Fig. 4b). To further confirm this, purified streptavidin was added to TLM-carrier capsids, and the mixture was loaded on a Ni-chelating column. As streptavidin was produced as a hexa-his-fusion protein, it is retained on the Ni-chelating column. If the TLM-carrier capsid binds to streptavidin, it should be retained as well and after elution be detectable in the respective fractions. Transmission electron microscopy revealed the presence of the capsid carrier in the elution fractions, and that streptavidin-loaded TLM-carrier capsid have a higher diameter and thicker “walls” as compared to the unloaded particles observed in the previous experiments (Fig. 4c and Supplementary Fig. S3). This reflects the successful loading of streptavidin on the surface of the carrier capsid.

To characterize in details the binding of streptavidin to the strep-tag III presented on the spike tip of the TLM-core protein, the kinetic parameters of the interaction were determined by surface plasmon resonance (SPR) using a Biacore system. The TLM-capsid carrier was immobilized on a CM5-chip and the purified streptavidin was applied on the chip in five different concentrations (4.7–75.7 nM). Three different mathematical models were used to fit the experimental data: The 1:1 binding model, the heterogeneous ligand model, and the bivalent analyte model. In the 1:1 binding model (A + B → AB) one molecule streptavidin is supposed to bind to one strep-tag molecule presented on the immobilized core protein. Although this model was previously applied to describe the interaction between streptavidin or other biotin-binding molecules and immobilized biotin^44, 45^, the fitting obtained from this model was poor compared to the other two models (Fig. 4d and Supplementary Fig. S3), therefore, the interaction parameters obtained from the other two models were used. In the bivalent analyte model (A + B → AB, AB + B → AB_2_), each streptavidin molecule is supposed to bind to one or two strep-tags on the carrier capsid. Binding to two strep-tags will strengthen the overall binding, and this could explain the slow off-rate values which were measured (Tables 1 and 2). In Table 1, the kinetic parameters for the interaction between streptavidin (or Strep-Tactin) and different ligands, which were described in previous studies, were compared to those values obtained in our study. The amino acid sequence for the ligands which were mentioned in Table 1, are listed in Table 2.

**Table 1:** Kinetic parameters of the interaction between streptavidin and different binding ligands.

Protein	Ligand	On-rate (M^-^ s^−1^)	Off-rate (s^−1^)	*K*_D_ (M)	Reference
Streptavidin	Biotin	5.13 × 10^6^	2 × 10^−7^*	4 × 10^−14^	49, 50
Streptavidin	SBP-Tag	3.17 × 10^5^	4.85 × 10^−4^	1.53 × 10^−9^	45
Streptavidin	SBP-Tag2	3.38 × 10^5^	4.96 × 10^−4^	1.47 × 10^−9^	45
Streptavidin	Strep-tag	—	—	37 × 10^−6^	56
Streptavidin	Strep-tag II	—	—	13 × 10^−6^	48
Streptavidin	Nano-tag15	—	—	4 × 10^−9^	57
Streptavidin	Nano-tag9	—	—	17 × 10^−9^	57
Strep-Tactin	GFP-Twin-Strep (strep-tag III)	1.5 × 10^5^	17 × 10^−4^	11 × 10^−9^	51
Streptavidin^(HL)^	Strep-tag III	K_a1_ = 5.47 × 10^5^ K_a2_ = 3.97 × 10^5^	K_d1_ = 0.02616 K_d2_ = 0.001883	K_D1_ = 1.06 × 10^−7^ K_D2_ = 4.74 × 10^−9^	Our study
Streptavidin^(BA)^	Strep-tag III	K_a1_ = 1.46 × 10^5^ K_a2_ = 8.16 × 10^−5^ (RU^-^ s^-^)	K_d1_ = 0.02125 K_d2_ = 0.001256	**	Our study
Streptavidin-Ova^(HL)^	Strep-tag III	K_a1_ = 1.32 × 10^4^ K_a2_ = 3.56 × 10^5^	K_d1_ = 0.01267 K_d2_ = 7.0 × 10^−4^	K_D1_ = 9.57 × 10^−7^ K_D2_ = 1.97 × 10^−9^	Our study
Streptavidin-Ova^(BA)^	Strep-tag III	K_a1_ = 9.42 × 10^4^ K_a2_ = 4.28 × 10^−4^ (RU^-^ s^-^)	K_d1_ = 0.01043 K_d2_ = 8.23 × 10^−4^	**	Our study

*Value was calculated based on the off-rate and the *K*_D_ shown in the table. **The program used to analyze the data provides no *K*_D_ values when the bivalent analyte model is used to fit the data. HL: The heterogeneous ligand model was used, BA: The bivalent analyte model was used.

**Table 2:** The amino acid sequence of the ligands which were mentioned in the Table 1.

Ligand	Amino acid sequence
SBP-Tag	MDEKTTGWRGGHVVEGLAGELEQLRARLEHHPQGQREP
SBP-Tag2	GHVVEGLAGELEQLRARLEHHPQG
Strep-tag	AWRHPQFGG
Strep-tag II	WSHPQFEK
Nano-tag15	DVEAWLGARVPLVET
Nano-tag9	DVEAWLGAR
Twin-Strep (Strep-tag III)	WSHPQFEKGGGSGGGSGGSAWSHPQFEK
Strep-tag III (our study)	WSHPQFEKGGGSWSHPQFEK

In the heterogeneous ligand model (A + B1 → AB1 and A + B2 → AB2), the analyte is supposed to bind to two ligand species. These species could be different molecules or different sites on the same ligand molecule. The number of ligands or ligand sites proposed in this model was defined by the software used for data analysis and is limited to two, due to high complexity, in an equimolar ratio. After using this model, we obtained two sets of parameters. The first describes a strong binding interaction with slow off-rate, which we assume might reflect the specific binding between streptavidin molecules and the strep-tag III. The second accounts for a weak binding interaction with fast off-rate, which could reflect unspecific binding of streptavidin to the immobilized core protein, or to impurities (Tables 1 and 2). The values obtained from this model were close to those obtained from the bivalent analyte model and to those which were described previously for the binding between streptavidin and other ligands such as SBP-Tag (Table 1 and 2). In addition, using the heterogeneous ligand model, a dissociation constant for the strong binding interaction was determined to be 4.74 nM (Tables 1 and 2).

In another experiment, the binding specificity was tested by immobilizing streptavidin on a CM5-chip followed by applying the TLM-carrier capsid or unrelated proteins (lysozyme and ovalbumin). No specific binding was detected in the case of lysozyme and ovalbumin, whereas significantly higher binding was observed when TLM-carrier capsid was applied on the chip (Supplementary Fig. S3).

Taken together, these data indicate that the strep-tag III inserted into the spike tip of the TLM-carrier capsid functions as a high-affinity adapter for streptavidin.

### TLM-carrier capsids deliver their cargo into the cytoplasm of target cells

The data described above demonstrate the functionality of the adapter. The next question was whether the TLM-carrier capsids are indeed able to transfer cargo across the plasma membrane into cells. To address this point, Huh 7.5 cells were grown in the presence of streptavidin-loaded TLM-core for 60 min. Cells incubated with free streptavidin served as negative control. After fixation, the internalized TLM-core and streptavidin protein were visualized by core- and streptavidin-specific antibodies, respectively. To ensure that indeed the intracellular components were analyzed, actin filaments were stained with TRITC-phalloidin.

Confocal laser scanning microscopy (CLSM) revealed a cytosolic staining of both, core and streptavidin, for the cells incubated with streptavidin loaded on the TLM-carrier capsids (Fig. 4e). On the contrary, for cells incubated with free streptavidin, no significant intracellular streptavidin-specific staining was detectable. These results demonstrate the capacity of TLM-carrier capsids to translocate their surface-loaded cargo (here streptavidin) across the plasma membrane into the cytoplasm of target cells.

### Characterization of TLM-carrier capsid loaded with ovalbumin as a model antigen

**Figure 5:**
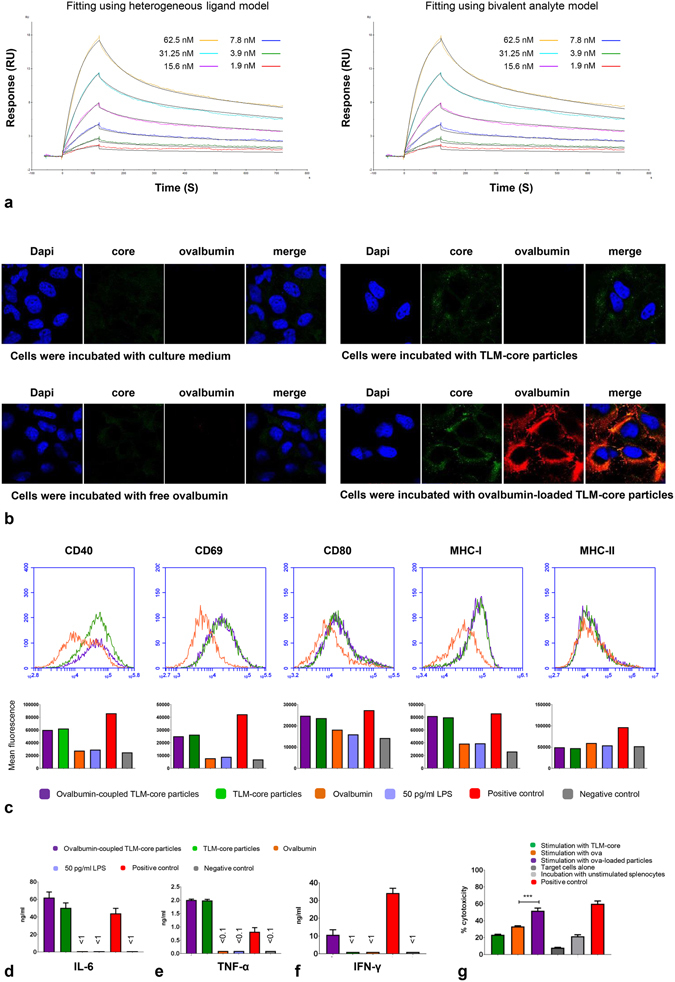
Ovalbumin as a model antigen for the induction of an antigen-specific CD8^+^ T-cell response. (**a**) Sensorgrams of SPR analysis of the interaction between streptavidin-ova and strep-tag III. “Heterogeneous ligand” or “bivalent analyte” models were used. Experimental results are drawn in different colors and theoretical curves in black. For further details, see Supplementary Fig. S4. (**b**) CLSM pictures of Huh 7.5 cells which were incubated with TLM-carrier loaded with streptavidin-Ova, TLM-core, streptavidin-Ova, or medium. Internalized proteins were stained with HBcAg-specific (green) and streptavidin-specific antibody (red). Nuclei were stained with DAPI (blue). (**c**) FACS analysis of BMDCs activation. BMDCs were incubated with 10 µg/ml TLM-carrier capsid loaded with 2.3 µg/ml streptavidin-Ova (molar ration 1:0.1), 10 µg/ml unloaded TLM-carrier capsid, 2.3 µg/ml free streptavidin-Ova, culture medium (negative control), or culture medium containing 1 µg/ml LPS (positive control). In addition, cells were incubated with the maximum LPS amount (50 pg/ml) which is possibly presented in the TLM-core sample. Expression of activation markers was analyzed by flow cytometry. The lower panel represents the corresponding mean fluorescence intensities of the measurements in the upper panel. The culture supernatant from the experiment described in c was collected, and the amount of secreted IL-6 (**d**) and TNF-α (**e**) was determined by ELISA. (**f**) Assessment of CD8^+^ T-cells activation by Ova-loaded TLM-carrier capsids. BMDCs were prepared and co-cultured with splenic CD8^+^ T-cells isolated from OT-1 transgenic mice in the presence or absence of the proteins (like in c). Culture medium or culture medium contains 4 mg/ml natural ovalbumin served as negative and positive controls, respectively. Secreted IFN-γ was measured by ELISA. (**g**) Assessment of the killing activity of Ova-specific CD8^+^ T-cells. Splenocytes were stimulated with proteins like in c. Culture medium or culture medium containing 5 µg/ml SIINFEKL peptide served as negative and positive controls, respectively. Cells were then incubated with target cells. The killing activity was assessed by flow cytometer and presented as % percentage. The experiment was done in triplicate (for d, e, f, and g). ***P < 0.001. The bars in the figures represent SD. For further data see Supplementary Fig. S5.

The experiments described above show that TLM-carrier capsids can be efficiently loaded via the strep-tag adapter and that cargo is indeed translocated across the plasma membrane. The resulting question was whether the system can be really used for the induction of a T-cell response. To investigate this, ovalbumin was chosen as a well-characterized model antigen^46^. Ovalbumin was produced as a fusion protein with streptavidin and a hexa-his tag in a eubacterial expression system and purified by affinity chromatography on a Ni-chelating column (Supplementary Fig. S4). The purity and identity of the isolated proteins were investigated by Coomassie-stained SDS-PAGE and Western blot analysis (Supplementary Fig. S4). The functionality of the purified and refolded protein was demonstrated by non-denaturing SDS-PAGE, showing the tetramerization of the fusion protein and by gel filtration, demonstrating the biotin binding capacity (Supplementary Fig. S4). To characterize in detail the binding of the streptavidin-Ova fusion protein to the TLM-carrier capsid, surface plasmon resonance (SPR) was performed. The TLM-core protein was immobilized on a CM5-chip and the purified streptavidin-Ova fusion protein was applied on the chip in six different concentrations (1.9–62.5 nM). After data analysis, the 1:1 binding model resulted in poor fitting of the experimental data compared to the bivalent analyte and heterogeneous ligand models (Fig. 5a and Supplementary Fig. S4). Using these two models to interpret the data, similar on- and off-rates were obtained (Tables 1 and 2). Fitting the experimental data with the heterogeneous ligand model resulted in two sets of parameters describe a strong and a weak binding interactions which could reflect a specific and nonspecific interaction, respectively. The *K*_D_ for the strong binding interaction was determined here to be 1.97 nM (Table 1 and 2). Moreover, using the off-rate value, the dissociation half-life (*t*_1/2_) of the strong binding interaction was determined to be 16.5 min using the following formula: *t*_1/2_ = ln_2_ ( = 0.693)/off-rate.

To investigate whether the streptavidin-Ova fusion protein bound to the surface of the TLM-carrier capsid can be translocated across the plasma membrane, Huh 7.5 cells were grown in the presence of Ova-loaded TLM-carrier capsid for 60 min. As controls, cells were incubated either with free streptavidin-Ova fusion protein, free TLM-carrier capsids, or culture medium. After fixation and staining with Ova- and core-specific antibodies, the cells were analyzed by CLSM. The confocal microscopy shows that in contrast to the free streptavidin-Ova, the streptavidin-Ova fusion protein loaded to the surface of the TLM-carrier capsids translocates across the plasma membrane into the cell (Fig. 5b).

Taken together, these results indicate that the TLM-carrier capsid can be efficiently loaded with the streptavidin-Ova fusion protein that serves as model antigen and that the antigen cargo is translocated by the TLM-carrier capsid into the cytoplasm of the target cells.

### Ovalbumin-loaded TLM-carrier capsids induce strong activation of bone marrow-derived dendritic cells

To investigate the capacity of Ova-loaded TLM-carrier capsids to induce activation of dendritic cells, bone marrow-derived murine dendritic cells (BMDCs) were incubated with Ova-loaded TLM-carrier capsids. As controls, cells were incubated with free streptavidin-Ova, free TLM-carrier capsids, culture medium (negative control), or 1 µg/ml LPS (positive control). All proteins used in this experiment were subjected to the LPS removal protocol, and the final LPS content was below 5 pg/µg protein.

Dendritic cells activation was assessed by FACS analysis to measure the expression of maturation markers (CD40, CD69, and CD80) and MHC class I and II molecules on the surface of the dendritic cells (Fig. 5c). The incubation of dendritic cells either with unloaded or with Ova-loaded TLM-carrier capsid led to an enhancement in the expression of all investigated markers except MHC class II molecules. Very low or no activation was observed when the cells were incubated with free streptavidin-Ova. These results indicate that there is a general adjuvant effect of the TLM-carrier capsid leading to an activation of DCs, and this effect persists even if the particles are loaded -on their surface- with ovalbumin. This is in accordance with a previous report that describes an adjuvant effect triggered by the HBV capsid^35^.

Activation of dendritic cells under these conditions was confirmed by measuring the secretion of IL-6 and TNF-α in the supernatant by ELISA. The amount of secreted IL-6 and TNF-α was clearly increased if the cells were incubated with TLM-carrier capsid or Ova-loaded TLM-carrier capsid, while free ovalbumin does not affect the amount of secreted IL-6 or TNF-α (Fig. 5d,e). This adjuvant effect of the HBV capsid was previously reported^35^. Taken together, these data indicate that the TLM-carrier capsid *per se* has a general adjuvant effect leading to an activation of DCs that supports the specific immune response.

### Ovalbumin-loaded TLM-carrier capsids induce strong specific activation of OT-I-derived CD8^+^ T-cells

The experiments described above have demonstrated that the cell permeability mediated by the TLM-core carrier capsid enables transfer of the ovalbumin cargo into the cytoplasm where a direct (immuno-)proteasomal processing can occur. Therefore, it was investigated whether the coupling of Ova to the surface of the TLM-carrier capsids indeed leads to an enhanced presentation of Ova-specific peptides and thus an enhanced activation of the Ova-specific CD8^+^ T-cells. To study this, murine BMDCs (from C57BL/6 N mice) and splenic CD8^+^ T-cells (from OT-I mice expressing a transgenic CD8^+^ T-cell receptor for MHC class I-restricted peptide OVA_257–264_) were co-cultured in the presence of Ova-loaded TLM-carrier capsid. As controls, cells were incubated with free streptavidin-Ova or free TLM-carrier capsid. Incubation with culture medium served as additional control. The activation of the splenic Ova-specific CD8^+^ T-cells was assessed by measuring the amount of secreted IFN-γ in the supernatant by ELISA 72 h after incubation. ELISA measurements revealed at least a 10-fold enhancement in the activation upon the incubation with Ova-loaded TLM-carrier capsid, compared to the activation induced by free streptavidin-Ova (Fig. 5f). To investigate whether the coupling process of streptavidin-Ova to the surface of the carrier capsids is crucial for the observed activation, recombinant ovalbumin, which lacks streptavidin and thus does not specifically bind to the capsid carrier, was mixed with the TLM-carrier capsids and applied in the co-culture experiment. In this case, a basal activation was observed that is similar to the activation triggered by the unloaded carrier capsid, (Supplementary Fig. S5). These results demonstrate that a simple mixture of the proteins is not enough, and a specific coupling of the antigens is important for the observed activation. To investigate the relevance of the cell permeability in the observed activation, ΔTLM-carrier capsid, which lakes the translocation motif, was applied. Coupling of streptavidin-Ova to the ΔTLM-carrier capsid led to enhancement of the amount of secreted IFN-γ (Supplementary Fig. S5), and this was expected since the capacity of the HBV capsid-derived VLPs to activate dendritic cells and enhance the activation of antigen-specific CD8^+^ T-cells was already described^20–22, 35^. However, more pronounced enhancement of the amount of secreted IFN-γ was observed when streptavidin-Ova was coupled to the TLM-carrier capsid (Supplementary Fig. S5).

The resulting question was whether the activation of CD8^+^T-cells by ovalbumin, loaded to the TLM-carrier capsid, leads to an enhanced cytotoxicity of the Ova-specific CD8^+^T-cells.

To address this, splenocytes were isolated from OT-I mice and stimulated with Ova-loaded TLM-carrier capsid for three days. As controls, splenocytes were stimulated with either free ovalbumin or free TLM-carrier capsids. Incubation with culture medium served as negative control. Stimulated splenocytes were incubated with CFSE-labelled and SIINFEKL peptide-pulsed EL4 cells as target cells. The killing activity of the stimulated splenocytes was assessed by measuring the percentage of the dead target cells. Significant higher killing activity (*P* = 0.0006) was found when splenocytes were stimulated with Ova-loaded TLM-carrier capsids as compared to stimulation with free ovalbumin (Fig. 5g). When recombinant ovalbumin (which lacks streptavidin) was mixed with the TLM-carrier capsids, a slight enhancement of the killing activity was observed (Supplementary Fig. S5). This could be due to an unspecific binding of ovalbumin on the capsid carrier. However, much more prominent and significant strengthening of the cytotoxicity was observed when streptavidin-Ova was loaded on the TLM-carrier capsids and applied in this experiment (Supplementary Fig. S5). This underlines again the importance of the coupling process for the observed increase. Moreover, weaker strengthening of the cytotoxicity was observed when streptavidin-Ova was coupled to the ΔTLM-carrier capsid as compared to TLM-carrier capsids (Supplementary Fig. S5). This proves the relevance of the cell permeability for the observed enhancement of the killing activity.

**Figure 6:**
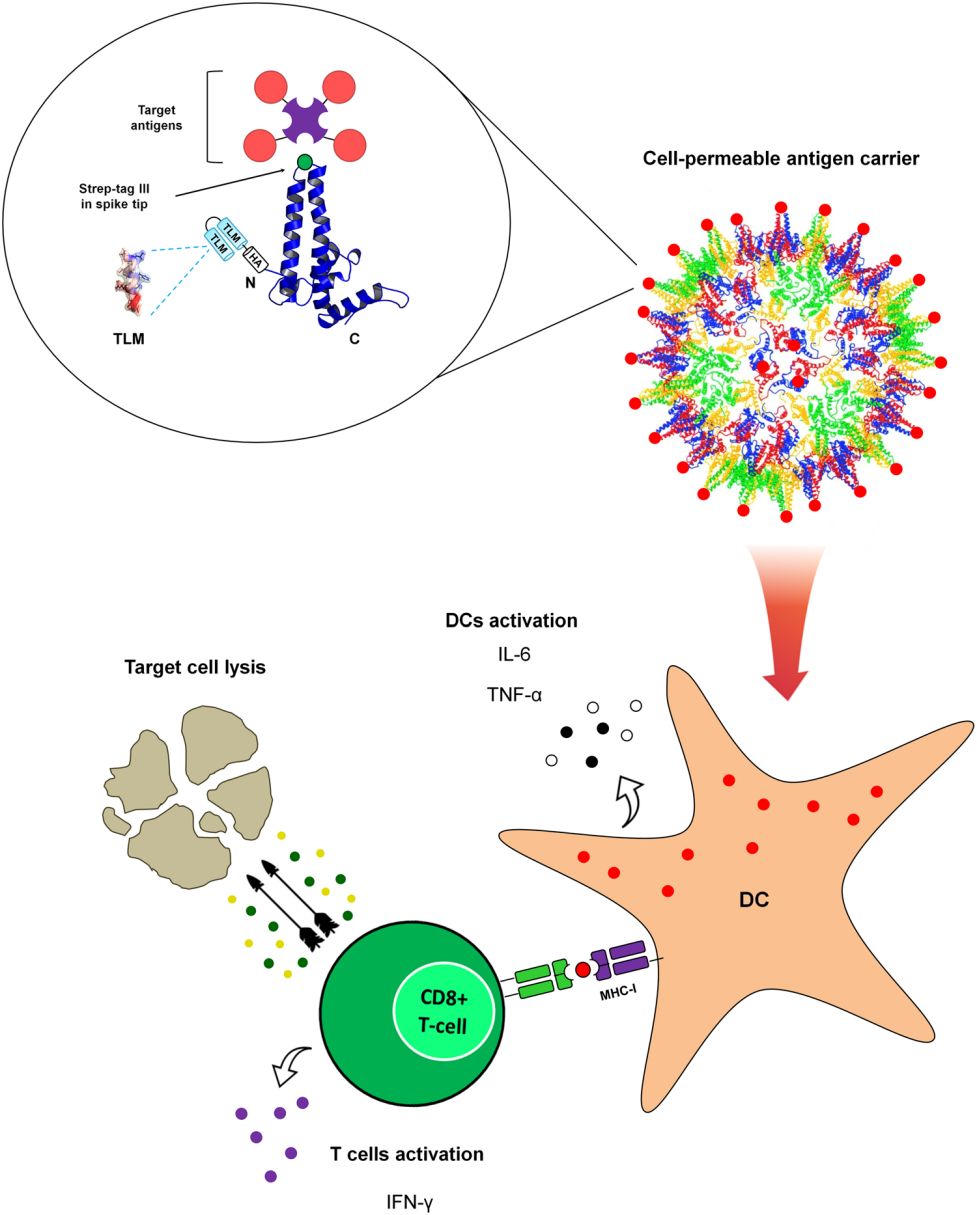
Schematic representation of the TLM-carrier capsid-based approach for induction of a robust CTL-response. (The schematic representation of the HBV core monomer and particle was modified from Wynne *et al*.^8^).

Taken together, these data indicate that the TLM-carrier capsid can be efficiently loaded with antigens via the strep-tag that serves as an adapter. Due to the cell permeability of the carrier capsid, the cargo antigen is translocated across the plasma membrane into the cytosol in the DCs that finally triggers a robust activation of CD8^+^ T-cells (Fig. 6).

## Discussion

HBV capsid-derived VLPs are well characterized and widely investigated for their capacity to serve as a vaccine template for the presentation of heterologous peptides^7^. Presentation of antigens on the surface of HBV capsids allows their presentation with a high density and in an ordered pattern that favors the induction of a B-cell response^17, 18^. Eliciting a robust cytotoxic T-cell response requires the efficient delivery of antigen into APCs, finally leading to the induction of CD8^+^ CTLs.

Apart from the induction of a robust B-cell response, there is an increasing need for vaccines that enable in addition the induction of a robust T-cell response. The approach described in this study is based on membrane-permeable carrier capsids assembled by modified HBcAg. To enable the carrier capsids to be flexibly loaded with variable antigens, an adapter-based approach was developed. The data described above demonstrate that stable TLM-carrier capsids can be formed and can be loaded via a strep-tag, that serves as an adapter, with various antigens. The high stability of the HBV-derived capsids was previously described^42, 43^. *In vitro* assembly of the TLM-carrier capsid was carefully characterized in light of the previous work performed by the Zlotnick lab^41^. The loading of antigens on the surface of the TLM-carrier capsids was characterized by electron microscopy, and the carrier/cargo interaction was analyzed by SPR (Biacore).

Using the Biacore evaluation system, three different mathematical models were applied to fit the experimental data: the 1:1 binding model, the heterogeneous ligand model, and the bivalent analyte model. Inspection of residual plots, χ^2^ values, and U values indicates that the 1:1 binding model provides a poor fitting to the data in our study. In contrast, the other two models provide good fitting of the experimental data. The heterogeneous ligand model, which was used in our study, describes the binding of the analyte to two different ligands exist in an equimolar ratio, or two different sites on the ligand molecule. The number of sites and its relative populations were determined by the software which was used to analyze the data. Therefore, two sets of interaction parameters were obtained using this model. The first set describes a binding with high affinity (*K*_D_ = 4.74 × 10^−9^ for streptavidin and 1.97 × 10^−9^ for streptavidin-Ova) and a slow off-rate, which is supposed here to describe the specific interaction between streptavidin (or streptavidin-Ova) and the strep-tag III. The other set describes a binding with low affinity (*K*_D_ = 1.06 × 10^−7^ for streptavidin and 9.57 × 10^−7^ for streptavidin-Ova) and fast off-rate and might describe here unspecific interaction between streptavidin (or streptavidin-Ova) and the core protein, partially denatured core protein, impurities, or partially accessible strep-tag III ligand i.e. due to steric hindrance. This model could provide a suitable interpretation of the interactions occurring in our system. However, it is possible that more than two classes of ligands or ligand sites are involved but cannot be interpreted by this model which limits the number of sites to two, due to high complexity.

In the bivalent analyte model, each streptavidin molecule is supposed to bind to one or two ligands, which might be realistic since streptavidin tetramer can bind up to four ligands. Structural considerations reveal multiple interaction possibilities of the streptavidin with the strep-tags inserted in the spike tips. In each biotin-binding face in the streptavidin tetramer molecule, the two biotin-binding pockets are separated by ~ 20 Å^47^. In the core protein, which was used in our study, the tandem strep-tag was inserted in the spike tip domain via flexible linkers (with a maximum linker length of ~ 34 Å) which allow the strep-tag to have different orientation possibilities. This might allow two strep-tag molecules, which belong to two core monomers forming one dimer or belong to two neighboring dimers on the HBV core particle, to bind two biotin-binding pockets on the same streptavidin tetramer molecule (Supplementary Fig. S6). This indicates that the bivalent analyte model might provide a realistic model to interpret the interactions occurring in our system. Moreover, the previously reported *K*_D_ value for the binding interaction between strep-tag II and streptavidin^48^ is approximately the square root of the values obtained in our study for the strong binding interaction between strep-tag III and streptavidin (Table 1). This might corroborate the suitability of the bivalent analyte model in our system. However, as streptavidin is a tetramer, a tetravalent analyte model, which is not available due to high complexity, would describe more precisely the possible interactions.

Due to the high complexity of the possible interactions between streptavidin (tetramer) and the strep-tags that are present in the spike tip (dimer), and the multiple copies of the spike tips present on the surface of the capsid particle, both models might be limited to explain adequately the multiple interactions between tetrameric streptavidin molecules and the capsid template in our system. However, both models provide good fitting of the experimental data (Figs 4d and 5a) and similar interaction parameters (on- and off-rates) (Table 1 and 2). In light of this, we could not dismiss any of them, and we think that, despite the limitations described previously, both models might describe the possibly-occurring interactions in our system and thus can be used as two independent approaches to analyze the data leading to comparable results. However, although both models provided similar interaction parameters, applying each model leads to different interpretations regarding the number of bound molecules on the surface of each carrier capsid. Therefore, further investigation to determine the average number of the ligands on each core particle is required.

The calculated *K*_D_ values in this study indicate that the binding affinity between streptavidin and strep-tag III is in the range of nM which is much weaker than the one described for streptavidin and its natural ligand, biotin (*K*_D_ is in the range of fM^49, 50^), but is comparable to the previously reported results describing the affinity between streptavidin and other developed tags (Table 1 and 2). The *K*_D_ value of the interaction between strep-tag II and streptavidin was previously reported to be in the range of µM^48^. The enhanced affinity observed in our study might be due to the use of strep-tag III (=2xstrep-tag II) which was previously reported to bind Strep-Tactin with a *K*_D_ in the range of nM^51^.

The obtained interaction parameters were also used to calculate the dissociation *t*_1/2_ of the strong binding interaction between streptavidin-Ova and the carrier capsid. The calculated half-life was about 16.5 min, which means that about 94% of the bound streptavidin-Ova will dissociate after 1 h (=4x *t*_1/2_). However, our previous data showed that TLM-mediated internalization is a rapid process^36, 37^ ensuring that a sufficient amount of the antigen-loaded carrier capsids encounters the APC in a relatively short time. In addition, *ex vivo* pulsing of the dendritic cells could be used for delivering the antigen-loaded carrier capsid in APCs, which could help to overcome the short dissociation half-life.

In a previous study, it was described that fusion of the TLM-peptide to HBcAg leads to membrane-permeable capsids that can be used for gene transfer^36^. Our study shows that the membrane permeability of TLM-carrier capsids enables the transfer of cargo antigen, coupled via the streptavidin/strep-tag interaction, across plasma membranes. Using ovalbumin as model antigen, this platform was used to investigate whether this technique indeed can be used for the induction of a T-cell response.

Our results demonstrate that the TLM-carrier capsid *per se* triggers an activation of the DCs, shown here by upregulation of maturation markers and secretion of IL-6 and TNF-α. This activation, which was previously described for HBV capsids^35^, represents an advantage when the capsids are used as a vaccine template. In addition, this general activation of DCs by the TLM-carrier capsid was independent from ovalbumin. However, specific stimulation of Ova-specific CD8^+^ T-cells (IFN-γ secretion) and the following enhanced killing activity was mainly observed for the OVA-coupled TLM-carrier capsids, and not for the uncoupled capsids. This emphasizes the specificity of the CD8^+^ T-cell activation observed in our system and the relevance of the specific coupling of the antigen to the membrane-permeable carrier capsid. In light of this, we conclude that the membrane permeability of the carrier capsid facilitates the efficient delivery of the antigen-loaded capsid into the cytoplasm of DCs which, in parallel with the adjuvant effect of the carrier capsid, leads to the induction of CD8^+^ CTLs response and specific killing of the target cells.

The relevance of membrane permeability for the observed specific activation and enhancement of the killing activity was shown by using an HBV carrier capsid (ΔTLM-carrier), which lacks the TLM. Moreover, a simple mixture of the TLM-carrier capsid and the antigens that are not coupled to the carrier failed to drive comparable activation. This emphasizes the importance of the antigen coupling on the carrier surface in the observed results.

Taken together, the data described above demonstrate a proof of principle of a novel platform, based on membrane-permeable carrier capsids, as universal antigen carrier. However, for further characterization of the capsid template regarding *in vivo* stability, safety, compatibility, and efficacy, a suitable animal model is required and further investigations are necessary to clarify these points.

There is an increasing need of platform technologies for preventive and therapeutic vaccine development. The use of a universal platform, which can be efficiently loaded with various antigens, may help to shorten response times to emerging infections or novel pathogens. The carrier capsid can be easily produced in high amounts and can be characterized for a mock-up vaccine regarding all aspects relevant for an authorization. Based on a mock-up authorization^52^ of such a platform, the time-consuming process for market authorization of a vaccine could be shortened by an adaption for the specific antigen. In addition, the high flexibility of this approach could be relevant for personalized medicines if for example patient-specific tumor antigens have to be transferred into DCs.

In contrast to vaccine platforms based on live-attenuated viruses the active component in this approach is purified protein. This might be helpful for the acceptance of this approach and for an application in patients with a partial Immunosuppression, as no replication-(in)competent virus or viral nucleic acids are administered for immunization.

An advantage of the TLM-carrier capsid based approach described here is that the carrier can be easily produced in high amounts and efficiently assembled *in vitro* ^53^. Moreover it was shown that the interior of the capsid can be efficiently loaded with nucleic acids^36^. Therefore, the TLM-carrier capsid would have a further potential to enhance immune responses by loading an adjuvant such as CpGs of Poly (I:C)^54^.

With respect to preventive vaccines, the induction of a robust T-cell response, in addition to the B-cell response, might be relevant for the control of pathogens triggering poor B-cell responses and/or displaying a high genetic variability. In this case, the induction of robust CD8^+^ T-cell response could further contribute to prevent the onset of a productive infection by elimination of the infected cells and by an IFN-γ response.

In addition to preventive vaccines, there is a frequently unmet need for therapeutic vaccines. Examples are chronic HBV or HCV infection that are in many cases based on an insufficient CTL-response that prevents the efficient elimination of the infected cells and thereby the cure of the acute infection. In this case, boostering of the CD8^+^ T-cell response could contribute to eliminate the infected cells and thereby cure the chronic infection. Further applications could be the CTL-mediated destruction of tumor cells. As mentioned above the high flexibility of the adapter-based approach would allow individual loading of the carrier with patient-specific tumor antigens.

Taken together, the study described the proof of principle for a novel vaccine platform technology as summarized schematically in Fig. 6. As various antigens can be loaded via an adapter, this platform can be used to shorten the response time on novel emerging pathogens. The platform has the potential to trigger a robust CD8^+^ T-cell response, so it can be useful for the development of preventive and therapeutic vaccines.

## Material and Methods

Bacterial expression vectors were established by standard methods. For details, see *Supplementary Information*.

### Core protein purification and *in vitro* assembly to core particles

The bacterial expression vectors pET24d(+)_TLM-HA-core-strep-tag III (pTLMcoreStrep) and pET24d(+)_HA-core-strep-tag III (pΔTLMcoreStrep) were transformed into *Rosetta BL21[DE3] E*. *coli*. Protein purification was done using 5 ml strep-Tactin affinity column (GE Healthcare, Freiburg, Germany) on an ÄKTA chromatography system (ÄKTA purifier). The purified TLM-core protein was assembled to carrier particles *in vitro* by increasing NaCl concentration. The ΔTLM-core protein assembled spontaneously into core particles. For more details, see *Supplementary Information*.

### Purification of streptavidin and streptavidin fusion proteins

The bacterial expression vector pET21a-streptavidin-Alive^55^ (a gift from Alice Ting (Addgene plasmid # 20860)) and pET21a-streptavidin-Alive-his-ovalbumin were transformed into *BL21[DE3] E*. *coli*. Protein purification was performed under denaturing conditions using a Ni-laded HiTrap chelating column (GE Healthcare, Freiburg, Germany). For details, see *Supplementary Information*. Recombinant ovalbumin was kindly supported by Stefan Schülke, Paul-Ehrlich-Institut.

### LPS removal from the purified proteins

For LPS removal, Triton X-114 (Sigma-Aldrich Seelze, Germany) was added to the purified protein solution to a final concentration of 1%. After shaking at +4 °C for 20 minutes, phase separation was done by shaking at 25 °C for 10 minutes. Samples were then centrifuged for 10 minutes at 25 °C, 17000 × *g*. The upper (Triton X-114-poor) phase was collected, and Triton X-114 was added again to the same final concentration. Separation was repeated and the supernatant was dialyzed extensively against PBS for 3 days with changing the buffer every 12 h. The final LPS-depleted protein solution was concentrated, and endotoxin content was measured by limulus amebocyte lysate test (Charles River, Freiburg, Germany) according to the manufacturer’s instructions.

### Sucrose gradient density centrifugation

To investigate particle formation (Fig. 3a,b) the sample (1 ml) was laid at the top of a sucrose gradient formed by sucrose dissolved in TN150 buffer as following:

**Table:** 

Sucrose concentration (% w/v)	10	20	30	40	50	60	70
Volume (ml)	2	1	1	1	1	1	1

The tubes were then centrifuged for 18 h at 10 °C, 41000 rpm using SW 41 Ti rotor (Beckman Coulter, Krefeld, Germany). One ml-sucrose fractions were collected from the top of the gradient. For experiment in Fig. 4b, assembled TLM-carrier capsid (0.5 ml) were mixed with purified streptavidin (0.5 ml) and incubated for 60 minutes at +4 °C. The mixture was then laid on the surface of sucrose gradient formed by sucrose dissolved in TN150 buffer as following:

**Table:** 

Sucrose concentration (% w/v)	10	20	30	40	50	60
Volume (ml)	4	1	1	1	1	1

The tubes were then centrifuged for 20 h at +4 °C, 41000 rpm using SW 41 Ti rotor (Beckman Coulter, Krefeld, Germany). As a control, free streptavidin was laid on the sucrose gradient. One-ml sucrose fractions were collected from the bottom to the top of the sucrose gradient and analyzed by Western blot using HBcAg- (Merck Millipore, Darmstadt, Germany) and hexa-His-specific (Santa Cruz, Heidelberg, Germany) antibodies. Fractions 3 to 9 were shown in the blots. The sucrose density gradient centrifugation was performed on a preformed gradient. The preformed gradient was built up by sequential layers starting from the higher (bottom) to the lower (top) density with 10% decrease of the sucrose content between each layer and the next one. The sample was applied at the top, followed by ultracentrifugation.

### Surface plasmon resonance (SPR)

Surface plasmon resonance (SPR) was performed using a Biacore T200 (GE Healthcare, Freiburg, Germany). For details, see *Supplementary Information*.

### Transmission electron microscopy (TEM)

Transmission electron microscopy (TEM) was performed by negative staining according to standard procedure: Twenty μl of the sample was applied on carbon coated and glam discharged formvar grids for 5–10 min at room temperature, followed by two washing steps with 50 µl ddH_2_O. Fifteen μl of 2% uranyl acetat (dissolved in ddH_2_O) was then applied on the grid for 10 sec at room temperature. The grid was let to dry and examined by TEM (EM 109, Zeiss, Jena, Germany).

### Mice

C57BL/6 N (B6) mice (Charles River, Freiburg, Germany) and OT-I mice (Jackson Laboratories, ME, USA) which express a transgenic CD8^+^ T-cell receptor for MHC class I-restricted OVA_257–264_, were bred at the animal facility of the Paul-Ehrlich-Institut. The mice were housed under specified pathogen-free conditions. Breeding of the mice was performed according to German animal protection law and had been approved by the local veterinary authorities of Darmstadt, Germany.

### Bone marrow isolation

Bone marrow isolation, generation of bone-marrow derived dendritic cells (BMDCs), assessment of BMDC activation, and assessment of CD8^+^ T-cell activation are described in detail in the *Supplementary Information*.

### *In vitro* killing assays

*In vitro* cytotoxic T lymphocyte (CTL) killing assay was analyzed by flow cytometry using BD Accuri C6 flow cytometer and software (BD, Heidelberg, Germany). For details, see *Supplementary Information*.

### Statistical analyses

Results are described as means ± standard deviation (SD). The significance of the results was analyzed by a ratio t test (two-tailed) using GraphPad Prism software. The bars in the figures represent SD.

## Electronic supplementary material

supplementary information
