# Supplementary material for: Cell-permeable capsids as universal antigen carrier for the induction of an antigen-specific CD8+ T-cell response
Source: Sci Rep. 2017 Aug 29;7:9630. doi: 10.1038/s41598-017-08787-0 (PMC5575276; doi:10.1038/s41598-017-08787-0)
Supplement: Supplementary file 1 — supplementary information [file 41598_2017_8787_MOESM1_ESM.pdf]

Cell-permeable capsids as universal antigen carrier for the induction of an antigen-specific CD8$^+$ T-cell response

Sami Akhras[1], Masako Toda[2], Klaus Boller[3], Kiyoshi Himmelsbach[1], Fabian Elgner[1], Marlene Biehl[1], Stephan Scheurer [2], Meike Gratz[1], Stefan Vieths [2] and Eberhard Hildt[1, 4,*]

[1-3)] Paul-Ehrlich-Institut, 63225 Langen, Germany, [1)] Dept. of Virology, [2)] Dept. of Allergology, [3)] Dept. of Immunology, [4)] German Center for Infection Research (DZIF), 38124 Braunschweig, Germany

*Correspondence should be addressed to E.H (eberhard.hildt@pei.de). Eberhard Hildt. Address: 63225 Langen, Germany. Tel: 0049 6103 77 2140. Fax: 0049 6103 – 77 1273

Supplementary Information

Supplementary figures

Supplementary Figure S1

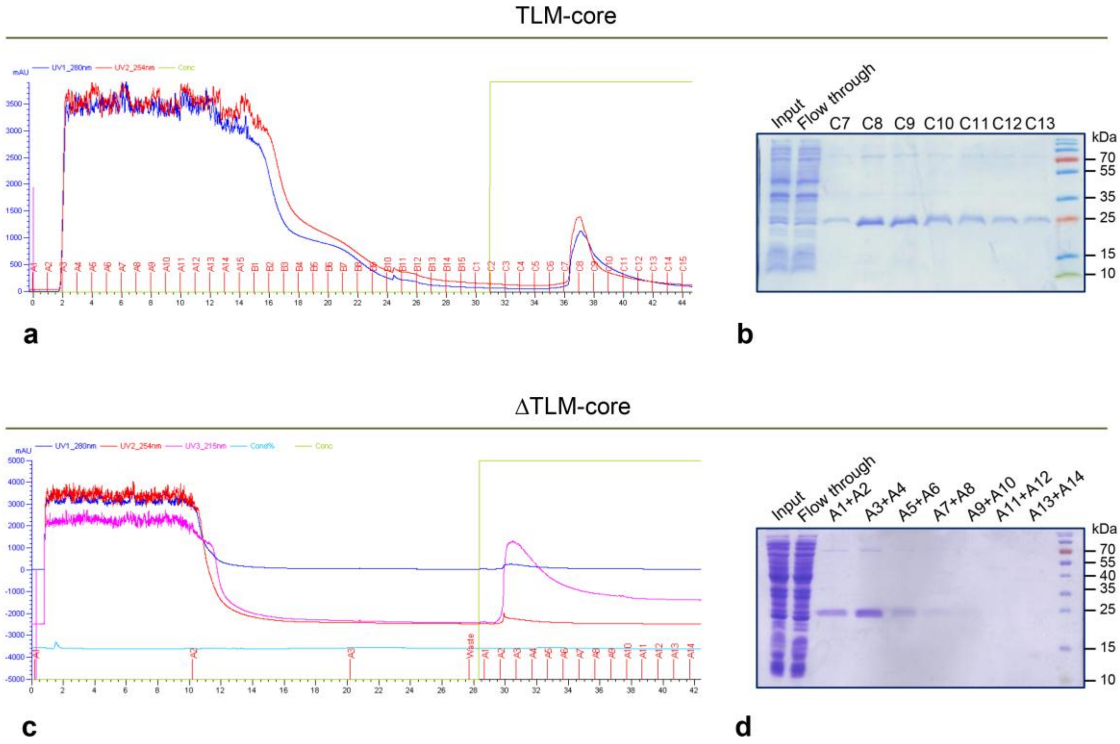

Figure S1. Purification of TLM-core and of ∆TLM-core proteins. Chromatogram of the strep-Tactin affinity purification of the TLM-core **(a)** and ∆TLM-core **(c)** proteins which were produced in *E. coli*. Selected fractions after the strep-Tactin purification were analyzed by Coomassie stained SDS-gel (**b** and **d**). The theoretical molecular weights of TLM-core protein and ΔTLM-core protein are about 25.4 and 24.5 kDa, respectively. TLM, translocation motif.

Supplementary Figure S2

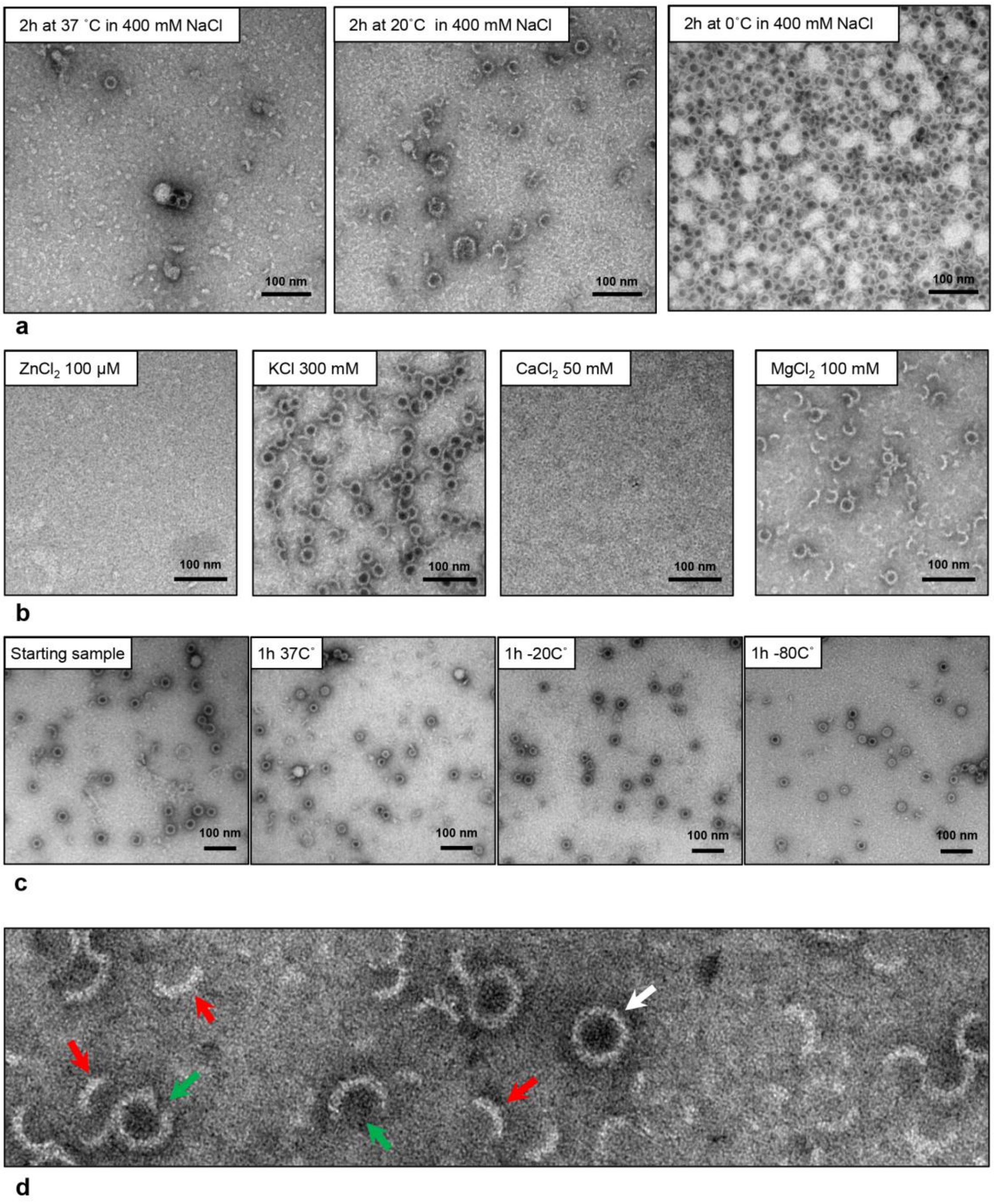

Figure S2. Assembly optimization of the TLM-core particles and the stability of the assembled particles Purified TLM-core protein (400 µg/ml = 15.7 µM) was assembled by incubating for 24 h in PBS contains 400 mM NaCl at 37 ˚C, 20 ˚C, and 0 ˚C (**a**) or by incubating for 24 hours at 0°C in PBS contains the indicated concentrations of $ZnCl_2$, KCl, $CaCl_2$, or $MgCl_2$ (**b**). Assembly products were scanned by TEM. (**c**) Purified TLM-core particles (2.4 mg/ml = 94.5 µM) were incubated for 1 hour at 37 ˚C, -20 ˚C and -80 ˚C and the effect on the particles integrity was analyzed by TEM. **(d)** High magnification of the assembly product in PBS buffer contains 660 mM NaCl. Fully assembled core particle, partially completed core particles, and small parts of core particles were indicated by white, green and red arrows, respectively (negative staining, scale bar = 100 nm).

Supplementary Figure S3

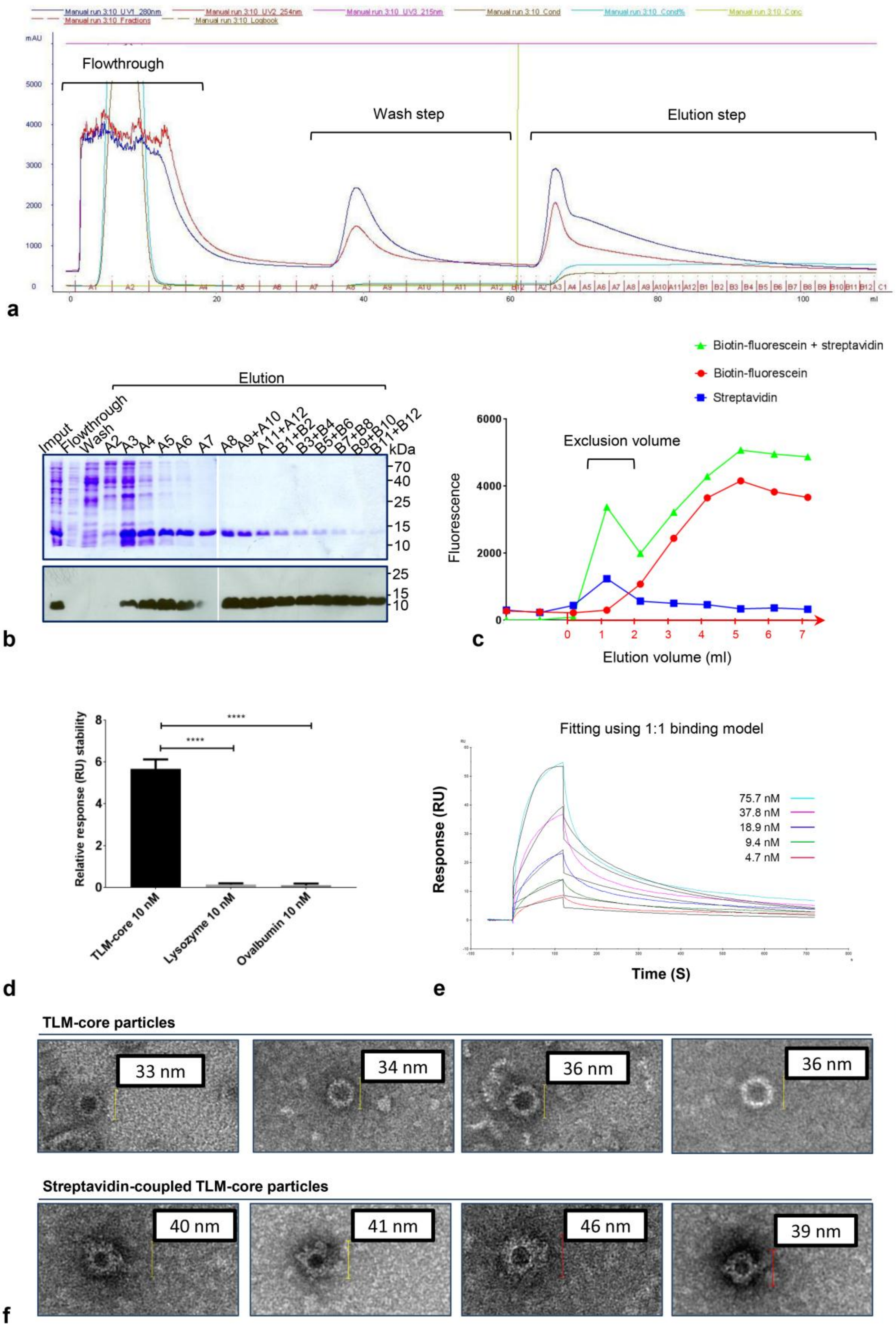

Figure S3. Purification and loading of streptavidin on the surface of the TLM-carrier capsids. (a) Chromatogram of the Ni-NTA affinity purification of streptavidin under denaturing conditions. **(b)** Selected fractions were analyzed by Coomassie staining (upper image) and Western blotting using hexa-His-specific antibody (lower image). **(c)** Purified streptavidin (100 µg/ml = 6.9 µM) was mixed either with PBS contains biotin-fluorescein (13.6 µM) or with PBS and applied on HiTrap desalting column. As a control, biotin-fluorescein (13.6 µM) was applied alone on the column. Fluorescence intensities (the y-axis) of elution fractions were measured using TECAN reader for all elution fractions (the x-axis). (**d**) Biacore was used to test the specificity of the interaction between TLM-core and streptavidin which was immobilized on CM5-sensor chip. The binding of streptavidin was compared to the binding of two unrelated proteins. ****, $P < 0.0001$. The bars in the figures represent SD. (**e**) Sensorgrams of the of SPR analysis of the interaction between streptavidin and strep-tag III on the surface of the TLM-carrier capsid. TLM-carrier was immobilized on a CM5-sensor chip and streptavidin was injected in five different concentrations. The "1:1 binding" fitting model was used. The experimental results are drawn in different colors and the theoretical curves in black. The kinetic parameters obtained from this model were not used because of the poor fitting. The dissociation constant (K_D), on-rates, and off-rates of the interaction were calculated using the bivalent analyte and the heterogeneous ligand models (Table 1 and 2, Fig. 4). **(f)** TEM pictures of unloaded TLM-carrier capsid (upper panel) or streptavidin-loaded TLM-carrier capsid (lower panel).

Supplementary Figure S4

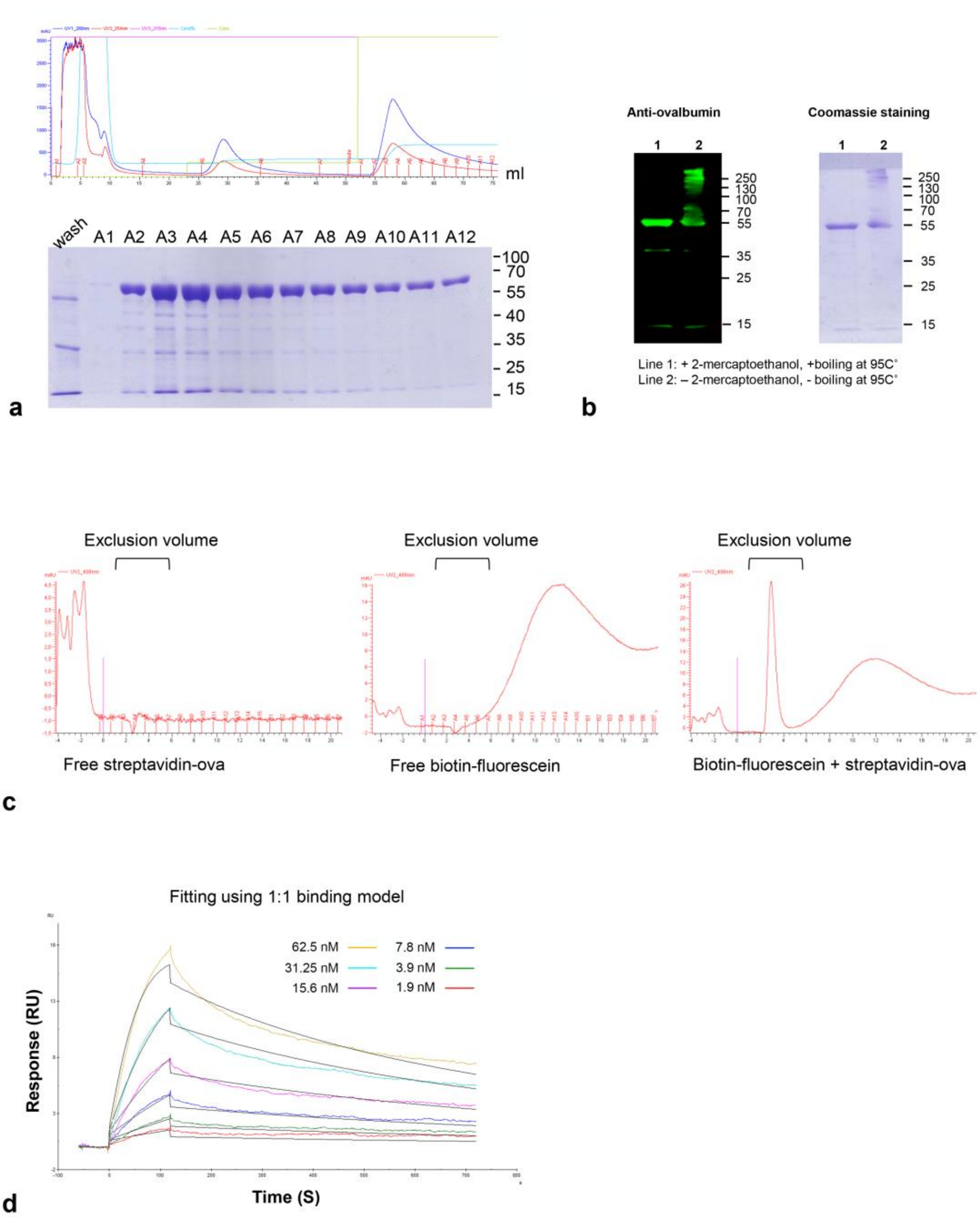

Figure S4. Purification and loading of streptavidin-Ova on the surface of the TLM-carrier capsids. (a) Chromatogram of the Ni-NTA affinity purification of streptavidin-Ova fusion protein under denaturing conditions (upper panel). Two-ml elution fractions were collected. Selected fractions after the purification were analyzed by Coomassie staining of SDS-PAGE (lower panel). The theoretical molecular weight of streptavidin-Ova fusion protein is about 58.4 kDa. (**b**) Denaturing and non-denaturing SDS-PAGE analysis of the purified streptavidin-Ova. SDS gels were either stained with Coomassie (right panel) or blotted and stained with ovalbumin-specific antibody (left panel) (for more details, see SI). **(c)** In order to investigate the biotin-binding capacity of streptavidin-Ova, two 5-ml HiTrap desalting columns were connected to each other and 10 µg biotin-fluorescein was injected alone or after previous mixing with 50 µg (= 0.86 µM) fusion protein. As a control, 50 µg fusion protein was injected alone. **(d)** Sensorgrams of the of SPR analysis of the interaction between

streptavidin-Ova and strep-tag III on the surface of the TLM-carrier capsid. TLM-carrier was immobilized on a CM5-sensor chip and streptavidin-Ova was injected in six different concentrations. The “1:1 binding” fitting model was used. Experimental results are drawn in different colors and theoretical curves in black. The kinetic parameters obtained from this model were not used because of the poor fitting. The dissociation constant (K_D), on-rates, off-rates, and the dissociation $t_{1/2}$ of the interaction were calculated using the bivalent analyte and the heterogeneous ligand fitting models (Table 1 and 2, Fig. 5).

Supplementary Figure S5

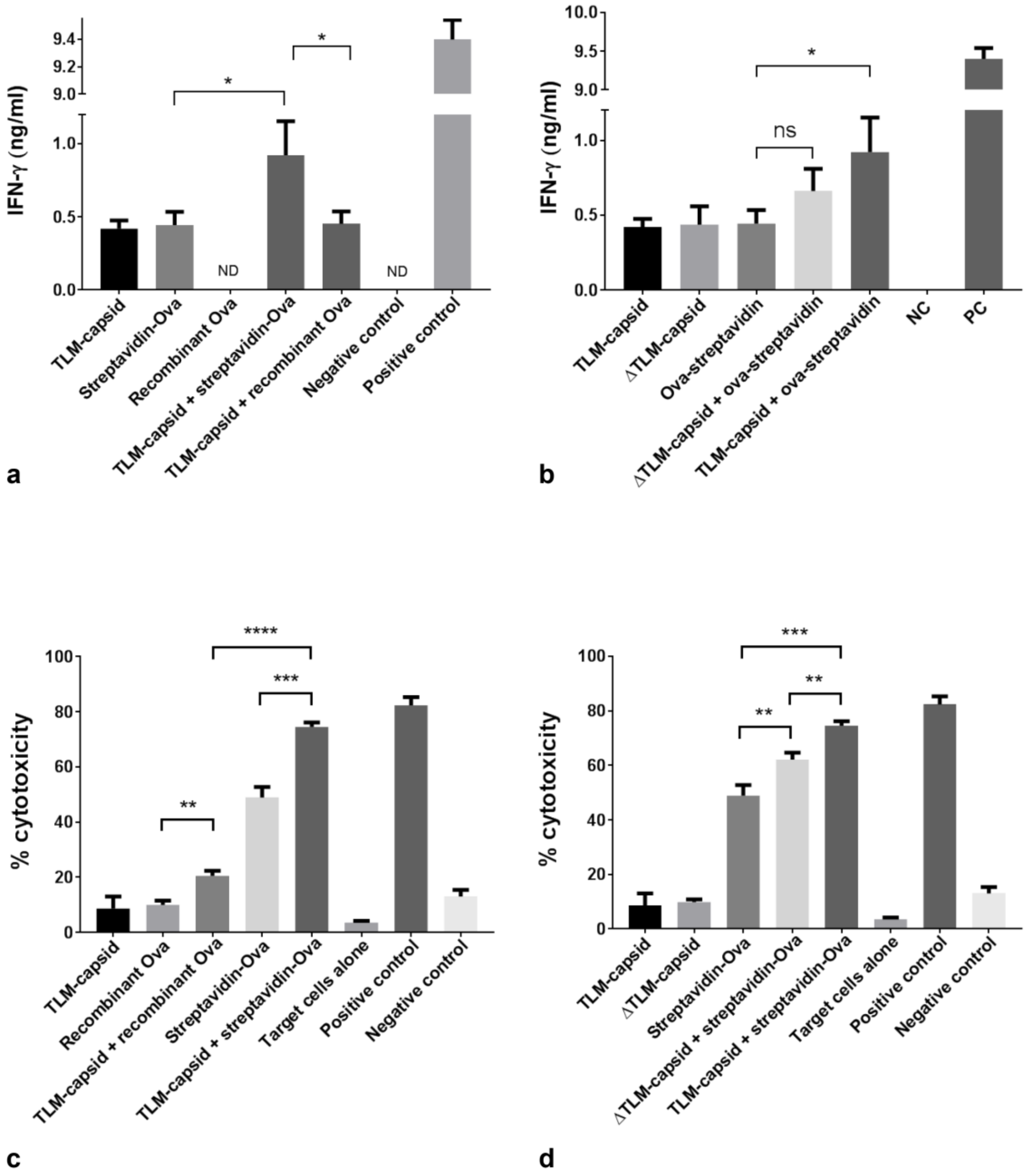

Figure S5. Induction of an antigen-specific CD8$^+$ T-cell response. (a) and **(b).** Assessment of CD8$^+$ T-cells activation by Ova-loaded TLM-carrier capsids. BMDCs were prepared and co-cultured with splenic CD8$^+$ T-cells isolated from OT-1 transgenic mice in the presence or absence of the proteins as following: 25 µg/ml TLM-carrier capsid, 25 µg/ml ΔTLM-carrier capsid, 5.7 µg/ml streptavidin-Ova, 4.25 µg/ml recombinant ovalbumin, 25

μg/ml ΔTLM-carrier capsid loaded with 5.7 μg/ml streptavidin-Ova (molar ratio 1:0.1), 25 μg/ml TLM-carrier capsid loaded with 5.7 μg/ml streptavidin-Ova (molar ratio 1:0.1), 25 μg/ml TLM-carrier capsid + 4.25 μg/ml recombinant ovalbumin (molar ratio 1:0.1). Incubation with culture medium or culture medium that contains 4 mg/ml natural ovalbumin served as negative and positive controls, respectively. Secreted IFN-γ was measured by ELISA. The experiment was done in triplicate. **(c)** and **(d)** Assessment of the killing activity of Ova-specific $CD8^+$ T-cells. Splenocytes were prepared and stimulated with proteins as following: 10 μg/ml TLM-carrier capsid, 1 μg/ml ΔTLM-carrier capsid, 2.3 μg/ml streptavidin-Ova, 1.7 μg/ml recombinant ovalbumin, 1 μg/ml ΔTLM-carrier capsid loaded with 2.3 μg/ml streptavidin-Ova (molar ratio 1:1), 10 μg/ml TLM-carrier capsid loaded with 2.3 μg/ml streptavidin-Ova (molar ratio 1:0.1), 10 μg/ml TLM-carrier capsid + 1.7 μg/ml recombinant ovalbumin (molar ratio 1:0.1). Different amounts of TLM- and ΔTLM-carrier capsids were used due to different ratios of assembled and non-assembled particles in each sample. Incubation with culture medium or culture medium containing 5 μg/ml SIINFEKL peptide served as negative and positive controls, respectively. Cells were then incubated with SIINFEKL-loaded and CFSE-labelled target EL4 cells. The killing activity was assessed by flow cytometry and presented as % killed cells from the total target cells (for more details see Supplementary Information). The experiment was done in triplicate. *, $P \leq 0.05$; **, $P \leq 0.01$; ***, $P \leq 0.001$; ****, $P \leq 0.0001$. The bars in the figures represent SD.

Supplementary Figure S6

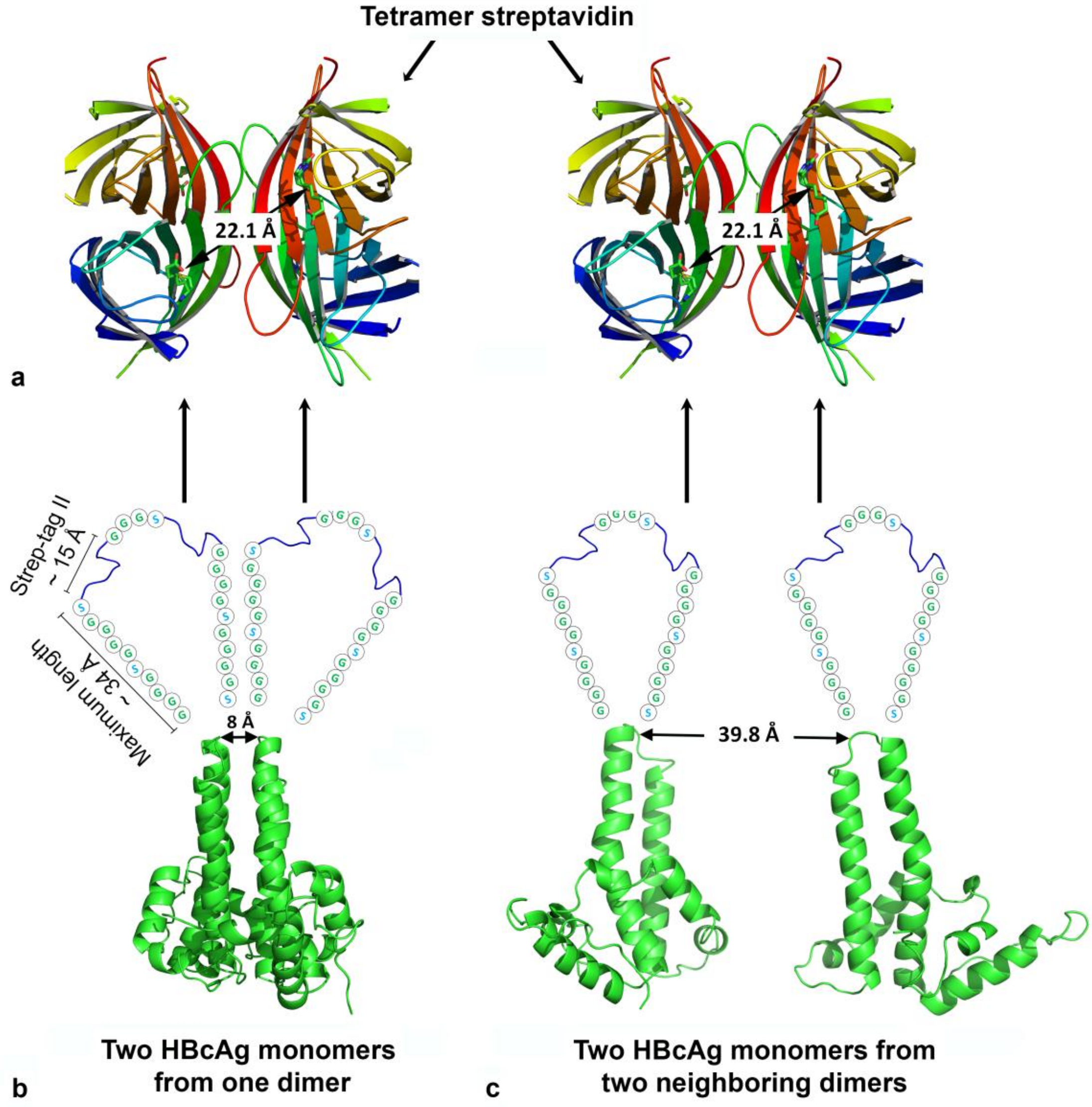

Figure S6. Schematic representation of **(a)** streptavidin-biotin complex (modified from Hyre *et al*., 2006[59]), and two HBcAg monomers belong to one dimer **(b)** or to two neighboring dimers **(c)** in the HBV capsid (modified from Wynne *et al*., 1999[8]). The adaptor, which is inserted in the spike tip and consists of strep-tag III (= 2 x strep-tag II (modified from Schmidt *et al*., 1996[57])) and two flexible linkers, is placed in the spike tip position. The distances are shown in Å and were measured by PyMOL software (The PyMOL Molecular Graphics System, Version 1.5.0.4 Schrödinger, LLC).

Supplementary materials and methods

Plasmids. For construction of a bacterial expression vector encoding for the TLM-core fusion protein harboring strep-tag III in the spike tip, the coding sequence of 2xTLM (ATG CCT GAC GGG ATT AGG TCG TTC ATC TCA TCG TTA CCC AGC GGC AGC CCC TTA TCG TCA ATC TTC TCG AGG ATT GGG GAC CCT) in addition to the coding sequence for the HA-tag (TAC CCA TAC GAC GTC CCA GAC TAC GCT) were inserted N-terminally to the coding sequence of the HBV capsid (ayw subtype, 1-151 aa) in the vector pET24d(+) using *NcoI* and *BamHI* as restriction sites. The coding sequence of strep-tag III (2xstrep-tag II) and two linkers (underlined) (*GAA TTC* GGT GGT GGT GGT AGC GGT GGC GGT GGT TCT TGG AGC CAT CCG CAG TTT GAA AAA GGT GGT GGT TCA TGG TCA CAC CCT CAG TTC GAG AAA GGC GGT GGT GGC TCA GGT GGT GGC GGT TCA *GAA TTC*) were inserted using the *EcoRI* (italic letters) restriction site between the coding sequence of the amino acids 78 and 82 in the HBV capsid coding sequence. The generated construct was named pET24d(+)_TLM-HA-core-strep-tag III (pTLMcoreStrep). The amino acid sequences of 1xTLM, 2xTLM, HA-Tag, strep-tag II, strep-tag III and the linker are: PDGIRSFISSLP, PDGIRSFISSLPSGSPLSSIFSRIGDP, YPYDVPDYA, WSHPQFEK, WSHPQFEKGGGSWSHPQFEK, GGGGSGGGGS. Another construct, pET24d(+)_HA-core-strep-tag III (pΔTLMcoreStrep), which lacks the 2xTLM motives and contains a C-terminal His-tag, was prepared using the first construct as template. For the expression of the streptavidin-Ova, the plasmid (streptavidin-Alive) was purchased from Addgene (pET21a-Streptavidin-Alive, #20860[55]). First, the stop codons were removed from the C-terminal using the primers: Streptav-alive-BamHI_fwd: AAA *GGA TCC* ACC TTC ATC GTT ACC GCT and streptav-alive-HindIII-minus stop_rev: AAA *AAG CTT* ATG GTG GTG ATG GTG ATG GGA AGC, and the generated vector was named pET21a-Streptavidin-Alive-His-minus-stop. Second, the coding sequence for wt-ovalbumin was amplified using ovalbumin expressing vector as template by the primers: Ovalbumin-HindIII_fwd: AAA *AAG CTT* GGC TCC ATC GGT GCA GCA AG, and ovalbumin-AvaI (XhoI)_rev: AAA *CTC GAG* AGG GGA AAC ACA TCT GC. The coding sequence of the restriction sites was written in italic letters. The PCR product were purified and digested with the corresponding restriction enzymes and ligated into the digested and dephosphorylated pET21a-Streptavidin-Alive-minus-stop vector. The final generated vector was named: pET21a-streptavidin-Alive-His-ovalbumin. Constructed plasmids were transformed into competent DH5-alpha *E. coli* for recombinant protein production.

Core protein purification and *in vitro* assembly into core particles. The bacterial expression vectors pET24d(+)_TLM-HA-core-strep-tag III (pTLMcoreStrep) and pET24d(+)_HA-core-strep-tag III (pΔTLMcoreStrep), which encode for TLM-core and ΔTLM-core proteins, respectively, were transformed into Rosetta BL21[DE3] *E. coli*. Transformed bacterial cells were grown in LB medium at 37 ˚C, and after reaching OD_{600} 0.4-0.5, IPTG (Thermo Scientific, Karlsruhe) was added to the bacterial culture to final concentration of 1 mM. The bacterial cells were incubated for further 4 h and then harvested. Pellets were resuspended in TN150 buffer (150 mM NaCl, 25 mM Tris-HCl, pH 8) containing protease inhibitors (leupeptin, aprotinin, PMSF, pepstatin (Applichem, Germany)) and 0.25 mg/ml lysozyme (Carl Roth, Karlsruhe, Germany). After 10 min incubation on ice, benzonase (Merck, Darmstadt, Germany) was added and sample was incubated for further 30 min at RT. Further disruption was done by sonication. Lysate was cleared by centrifugation at 20000 x *g* for 20 min at +4 ˚C. Protein purification was done using strep-Tactin affinity columns (GE Healthcare, Freiburg, Germany) on FPLC system (ÄKTA purifier), followed by desalting using the 5 ml HiTrap desalting column (GE Healthcare, Freiburg, Germany) according to the manufacturer's instructions. The identity and the purity of the purified proteins were analyzed by Western blot and Coomassie staining of SDS-gels, respectively. Regarding TLM-core protein, the purified core protein was assembled *in vitro* by increasing NaCl concentration. Different final NaCl concentrations, different assembly temperatures, different assembly periods, and different cations were tested and the proper assembly was checked by transmission electron microscopy (TEM). When the carrier capsids were used for DCs activation assay, $CD8^+$ T-cells activation assay, and for in vitro killing assay, Triton X-100 (Sigma-Aldrich Seelze, Germany) was added to the TN150 lysis buffer to final concentration of 1% (w/w), and the purified proteins were further dialyzed against PBS (pH 7.1) for 48 h. After dialysis, proteins samples were subjected to LPS removal protocol using Triton X-114.

Purification of streptavidin and streptavidin fusion proteins. The bacterial expression vector pET21a-streptavidin-Alive[55] (a gift from Alice Ting (Addgene plasmid # 20860)) and pET21a-streptavidin-Alive-his-ovalbumin were transformed into BL21[DE3] *E. coli*. Transformed bacterial cells were grown in LB medium at 37 ˚C, and after reaching OD_{600} 0.4-0.5, IPTG was added to the bacterial culture to a final concentration of 1 mM. The bacterial cells were incubated for further 4 h and then harvested. Inclusion bodies were extracted by resuspending the bacterial pellets in the lyses buffer: 100 mM Tris, pH 8 containing protease inhibitors (leupeptin, aprotinin, PMSF, pepstatin (Applichem, Germany), 100 g/l sucrose, 0.25 mg/ml lysozyme. The sample was incubated for 20 min at RT and cells were disrupted by sonification. Inclusion bodies were pelleted by centrifugation 20000 x *g* for 15 min at +4 ˚C. Pellets were washed five times with the same lysis buffer containing 1% triton X-100 (Sigma-Aldrich Seelze, Germany). An additional washing step with the lysis buffer containing 1 M NaCl was done before final washing with 100 mM Tris-HCl, pH 8 buffer. The purified inclusion bodies were dissolved in 6 M guanidinium hydrochloride buffer (6 M guanidinium-HCl, 100 mM $Na_2HPO_4.2H_2O$, 10 mM Tris, pH 8) for 2 h at RT, and the dissolved proteins were purified using Ni-laded HiTrap chelating column (5-ml HisTrap column, GE Healthcare) under denaturing conditions according to the manufacturer's instructions. The identity and the purity of the purified proteins were analyzed by Western blot and Coomassie staining of SDS-gels, respectively. Elution fractions which contain highly purified proteins were pooled and dialyzed against PBS (pH 7.1) subsequently containing 4, 2, 1, 0.5, and 0 M urea. When

streptavidin-Ova fusion protein was used for DCs activation assay, $CD8^+$ T-cells activation assay, and for in vitro killing assay, the purified proteins were subjected to LPS removal protocol using Triton X-114 (Sigma-Aldrich Seelze, Germany).

Non-denaturing SDS-PAGE. In order to check the correct folding and tetramerization of the purified streptavidin (Fig. 4a) and streptavidin-Ova (Fig. S4b) proteins, purified and refolded proteins were either mixed with loading buffer containing 2-mercaptoethanol and boiled at 95 ˚C for 10 minutes or mixed with loading buffer not containing 2-mercaptoethanol and applied without boiling on SDS-gel. The gel was then either stained with Coomassie or analyzed by Western blotting.

Surface plasmon resonance (SPR).

Surface plasmon resonance (SPR), the Biacore T200 equipment, was utilized to determine the kinetic parameters of the interaction between streptavidin, or streptavidin-Ova, and the strep-tag III, which is exposed on the surface of the TLM-core particles. TLM-core particles were covalently immobilized onto the surface of a CM5-sensor chip (FC-2) using the coupling reagents sulfo-NHS and EDC (amine coupling method) to a final immobilization level of 1833.1 response units (RUs). After the protein was immobilized, chip surface was treated with ethanolamine to deactivate the excess of reactive esters. To determine the kinetic parameters (on-rate and off-rate), dilution series of purified streptavidin or streptavidin-Ova (4.7 – 75.7 nM for streptavidin and 1.9 – 62.5 nM for streptavidin-ova) were injected in a multi-cycle experiment over the coated chip at flow rate of 30 µl/min using PBS (pH 7.1) containing 0.05% tween 20 and 3 mM EDTA as running buffer. The same running buffer was used to prepare the dilution series of the proteins. The increase and the decrease of the RUs measured during the association (120 s) and dissociation (600 s) phases, respectively, were used by the software (Biacore T200 control and Biacore T200 evaluation) to determine the kinetic parameters using different fitting models. Since harsh regeneration conditions would denature the immobilized proteins and no optimal regeneration conditions were found, we chose to perform the measurements without regeneration. Instead of regeneration, we applied a long dissociation time of 600 seconds and measured accumulating concentrations of the protein as recommended by the manufacturer. Performing SPR measurements without regeneration steps was also previously described.[56] A flow cell (FC-1), which was activated (sulfo-NHS and EDC) and deactivated (ethanolamine) without immobilization of a ligand, served as a reference cell for background correction. All measurements were performed at 25 °C. In addition, another CM5-sensor chip (only for Figure S3d) was coated with purified streptavidin at a final immobilization level of 2683.8 RUs and used to check the specificity of the interaction with Strep-tag III presented on the carrier capsid. The molecular masses of 25.4, 14.4, and 58.4 kDa were used for the monomer TLM-core, monomer streptavidin, and monomer streptavidin-Ova, respectively. Three different mathematical models were tested for the better fitting to the experimental data: The 1:1 binding model (single Langmuir isotherm), the heterogeneous ligand model, and the bivalent analyte model. The goodness of fit for each of these three different kinetic models was assessed by inspecting the residual plots, the χ^2 (Chi^2) value, and the U value (uniqueness value, only applicable in the case of 1:1 binding model). In addition, visual inspection of the fitted curves overlaid on the sensorgrams was performed. The 1:1 binding model ($A+B \rightarrow AB$) describes the binding of

one molecule streptavidin (or ova-streptavidin) to one strep-tag III molecule presented on the immobilized core particles. In the heterogeneous ligand model (A+B1→AB1 and A+B2→AB2) the analyte is supposed to bind two different ligands or two different positions in the immobilized ligand. The bivalent analyte model (A+B → AB, AB + B → AB_2) describes the situation where one analyte (streptavidin or ova-streptavidin) can bind one or two ligand molecules. Inspection of the mentioned values showed that the heterogeneous ligand model and the bivalent analyte model might describe the data better than the 1:1 binding model. Importantly, since fitting the data to a mathematical model does not provide a definitive evidence of the interaction mechanism, we do not exclude that a part of interaction might follow other mechanisms, where more complex mathematical models are required. A realistic model should include the possibility of the analyte (tetrameric streptavidin) to bind one, two, three, or four strep-tag III ligands immobilized on the chip. However, such a model will prove up to four on- and off-rates with four calculated K_D values, which raises the question about the reliability of the obtained data.

Confocal laser scanning microscopy (CLSM). Immunofluorescence staining was analyzed by the use of a confocal laser scanning microscope (CLSM; CLSM 510 Meta, Carl Zeiss, Jena, Germany) and ZEN 2009 software. To investigate the cell permeability, Huh 7.5 cells (the Huh7-derived cell line) were grown in a 24-well plate (1x 10^5 cells/well) in Dulbecco's modified Eagle's medium (DMEM; Sigma, Seelze, Germany) supplemented with 10% fetal calf serum (BioChrom/Merck, Darmstadt, Germany), 2 mM L-glutamine (BioChrom/Merck, Darmstadt, Germany), 100 U/ml penicillin (Carl Roth, Karlsruhe, Germany), and 100 µg/ml streptomycin (Carl Roth, Karlsruhe, Germany). For the experiment in figure 4e, the cells were incubated for 1 h in complete DMEM medium in the presence of 50 µg/ml TLM-core loaded with 37 µg/ml streptavidin or 37 µg/ml streptavidin. For the experiment in figure 5b, the cells were incubated in complete DMEM medium in the presence of 100 µg/ml TLM-core, 60 µg/ml streptavidin-Ova, or 100 µg/ml TLM-core loaded with 60 µg/ml streptavidin-Ova for 1 h. Cells were then washed three times with PBS (pH 7.1) and fixed with 4% formaldehyde for 10 min at RT and subsequently washed again with PBS (pH 7.1). Cells were then stained with HBcAg-specific antibody (Dako, Frankfurt, Germany) and streptavidin-specific antibody (Santa Cruz, Heidelberg, Germany). When indicated, actin filaments were stained with TRITC-phalloidin (Sigma-Aldrich, Seelze, Germany) and nuclei with DAPI (Carl Roth, Karlsruhe, Germany).

Bone marrow isolation generation of bone marrow-derived dendritic cells (BMDCs). Bone marrow cells were isolated from the femurs and tibia of C57BL/6N (B6) mice and erythrocytes were removed by red blood cell lysing buffer. Cells were cultured in RPMI 1640 (Thermo scientific, Karlsruhe, Germany) medium containing 10% fetal calf serum (BioChrom/Merck, Darmstadt, Germany), 1 mM sodium pyruvate (Thermo Scientific, Karlsruhe, Germany), 10 mM HEPES, 100 U/ml penicillin, 100 µg/ml streptomycin, 2 mM L-glutamin (BioChrom/Merck, Darmstadt, Germany), 0.1 mM 2-mercaptoethanol. For myeloid dendritic cells differentiation, recombinant mouse granulocyte macrophage colony stimulating factor ((rmGM-CSF), R&D Systems Wiesbaden-Nordenstadt, Germany) was added to the medium at a final concentration of 100 ng/ml. Medium was changed on day 3, 6, and 7. On day 8, cells were harvested for further experiments.

Assessment of BMDC activation. BMDCs (1 x 10^6 cells/ml) were stimulated with different protein combinations proteins (see figure ligands). Cells incubated only with culture medium or culture medium containing 1 µg/ml LPS served as negative and positive controls, respectively. After 18−20 h, culture supernatants were harvested to determine the concentration of secreted IL-6 and TNF-α by ELISA (eBioscience, Frankfurt a. M, Germany). In addition, cells were harvested and the expression levels of maturation markers on their surface were assessed by FACS analysis. *FACS analysis:* Cells were treated with anti-mouse CD16/CD32 mAb 2.4G2 (BD Pharmingen, Heidelberg, Germany) to block IgG receptors and then stained with FITC-conjugated anti-mouse CD40, CD69, CD86, MHC class I, or MHC class II molecule mAb (all from eBioscience, Frankfurt a. M, Germany). Additionally, the cells were stained with phycoerythrin (PE)-conjugated anti-mouse CD11b and allophycocyanin (APC)-conjugated anti-mouse CD11c mAbs (eBioscience, Frankfurt, a. M, Germany) to define the DC population. Fluorescence intensity in the cells was measured by flow cytometry using BD Accuri C6 flow cytometer and software.

Assessment of $CD8^+$ T-cell activation. $CD8^+$ T-cells were negatively isolated by $CD8^+$ T-cell isolation kit (Miltenyi Biotec, Bergisch-Gladbach, Germany) from spleen of OT-I mice according the manufacturer's instructions. Isolated $CD8^+$ T-cells (1 × 10^6 cells/ml) were then co-cultured with BMDCs (6.25 × 10^5 cells/ml) in the presence or absence of protein combinations (see figure ligands). Incubation with culture medium or culture medium contains 4 mg/ml natural ovalbumin served as negative and positive controls, respectively. Seventy two hours later, culture supernatants were harvested to determine the concentration of IFN-γ by ELISA (eBioscience, Frankfurt a. M, Germany).

***In vitro* cytotoxic T lymphocyte (CTL) killing assay.** *Splenocytes stimulation:* Splenocytes were prepared from OT-I mice and cultured (0.5 x 10^6 cells/ml in 96-well plate, round bottom) for 72 h in the presence of proteins (see figure ligands). Cells which were incubated with culture medium or culture medium containing 5 µg/ml SIINFEKL peptide (EMC Microcollections, Tübingen, Germany) served as negative and positive controls, respectively. *Target cells preparation:* EL4 lymphocytes were labeled with 5 µM CFSE (Abcam, Cambridge, UK) and pulsed with 30 µM SIINFEKL peptide (EMC Microcollections, Tübingen, Germany). Stimulated splenocytes (2.5 x 10^6 cells/ml) were co-cultured with CFSE-labeled and peptide-pulsed EL4 cells (2.5 x 10^5 cells/ml) for 4.5 h. Afterwards, cells were stained with propidium iodide (PI). The cells were gated for CFSE positive cells (target cells) and the viable cells were determined as negative PI-stained cells by FACS analysis. The killing activity was determined by calculating the percentage of the dead cells (PI positive cells) among all target cells (CFSE positive cells) and was shown as % cytotoxicity.
